# Electrographic Features of Spontaneous Recurrent Seizures in a Mouse Model of Extended Hippocampal Kindling

**DOI:** 10.1093/texcom/tgab004

**Published:** 2021-01-22

**Authors:** Haiyu Liu, Uilki Tufa, Anya Zahra, Jonathan Chow, Nila Sivanenthiran, Chloe Cheng, Yapg Liu, Phinehas Cheung, Stellar Lim, Yaozhong Jin, Min Mao, Yuqing Sun, Chiping Wu, Richard Wennberg, Berj Bardakjian, Peter L Carlen, James H Eubanks, Hongmei Song, Liang Zhang

**Affiliations:** 1 Departments of Neurosurgery, The First Hospital of Jilin University, Changchun, Jilin 130021 China; 2 Krembil Research Institute, University Health Network, Toronto, Ontario, Canada M5T 2S8; 3 Department of Electrical and Computer Engineering, University of Toronto, Toronto, Ontario M5S 3H5, Canada; 4 Department of Medicine, University of Toronto, Toronto, Ontario M2K 1E2, Canada; 5 Department of Physiology, University of Toronto, Toronto, Ontario M5S 1A8, Canada; 6 Department of Surgery, University of Toronto, Toronto, Ontario M5G 1X5, Canada

**Keywords:** brain slices, convulsion, epilepsy, ictal discharges, intracranial electroencephalograph

## Abstract

Epilepsy is a chronic neurological disorder characterized by spontaneous recurrent seizures (SRS) and comorbidities. Kindling through repetitive brief stimulation of a limbic structure is a commonly used model of temporal lobe epilepsy. Particularly, extended kindling over a period up to a few months can induce SRS, which may simulate slowly evolving epileptogenesis of temporal lobe epilepsy. Currently, electroencephalographic (EEG) features of SRS in rodent models of extended kindling remain to be detailed. We explored this using a mouse model of extended hippocampal kindling. Intracranial EEG recordings were made from the kindled hippocampus and unstimulated hippocampal, neocortical, piriform, entorhinal, or thalamic area in individual mice. Spontaneous EEG discharges with concurrent low-voltage fast onsets were observed from the two corresponding areas in nearly all SRS detected, irrespective of associated motor seizures. Examined in brain slices, epileptiform discharges were induced by alkaline artificial cerebrospinal fluid in the hippocampal CA3, piriform and entorhinal cortical areas of extended kindled mice but not control mice. Together, these in vivo and in vitro observations suggest that the epileptic activity involving a macroscopic network may generate concurrent discharges in forebrain areas and initiate SRS in hippocampally kindled mice.

## Introduction

Epilepsy is a chronic neurological disorder characterized by unprovoked or spontaneous recurrent seizures (SRS) and comorbidities. Temporal lobe epilepsy is the most common type of epilepsy seen in adult and aging populations and highly diverse in etiologies and electro-clinical manifestations (Engel Jr 1996; Ferlazzo et al. 2016). Currently, about one third of epilepsy patients are resistant to treatments with common antiepileptic drugs and only a small portion of these patients are candidates for surgery or other alternative treatments such as vagal nerve or deep brain stimulation. It is therefore imperative to understand the pathogenesis of epilepsy to yield more efficacious antiepileptic treatments.

Kindling through repetitive brief electrical stimulations of a limbic structure has long been used as a model of temporal lobe epilepsy (see reviews by Gorter et al. 2016; Löscher 2016; Sutula and Kotloski 2017). While kindling via the classic or standard protocol lasting a few weeks does not generally induce SRS, over or extended kindling that applies ≥100 stimulations over a period of up to a few months can induce SRS. Specifically, extended kindling induced SRS in monkeys (Wada et al. 1975; Wada and Osawa 1976), dogs (Wauquier et al. 1979), cats (Wada et al. 1974; Gotman 1984; Shouse et al. 1990, 1992; Hiyoshi et al. 1993), rats (Pinel and Rovner 1978a, 1978b; Milgram et al. 1995; Michael et al. 1998; Sayin et al. 2003; Brandt et al. 2004), and mice (Song et al. 2018b). Unlike commonly used models that induce acute status epilepticus by an application of kainite/pilocarpine or intensive brain electrical stimulation and then result in subsequent SRS (Mazarati et al. 1998; Dudek and Staley 2017; Gorter and van Vliet 2017; Henshall 2017; Kelly and Coulter 2017), SRS development following extended kindling is a slowly evolving process without initial status epilepticus (Aronica et al. 2017; Sutula and Kotloski 2017). As such, the extended kindling model may complement the status epilepticus and other SRS models (Navarro Mora et al. 2009; Walker et al. 2017) to study diverse epileptogenic processes relevant to human temporal lobe epilepsy (Engel Jr 1996; Ferlazzo et al. 2016).

The electroencephalographic (EEG) features of SRS have been characterized in kindled monkeys and cats via simultaneous intracranial recordings from multiple brain structures (Wada et al. 1975; Wada and Osawa 1976; Hiyoshi et al. 1993). In prefrontal or amygdala-kindled monkeys, focal motor seizures at the stage 2 of Racine scale (Racine 1972) featured unilateral discharges in hippocampal, prefrontal, and piriform cortical areas, and generalized seizures at the Racine stage 5 manifested concurrent bilateral discharges in multiple cortical and subcortical structures (Wada et al. 1975; Wada and Osawa 1976). In amygdala-kindled cats, focal motor seizures at the Racine stage 1–2 featured cortical discharges unilateral to kindled amygdala with minimal propagation to the subcortical recording sites, and generalized stage 5 motor seizures resulted from discharges that initially arose from kindled or unstimulated amygdala and ipsilateral dorsomedial thalamus and globus pallidus and then spread with evident time delays to other bilateral structures (Hiyoshi et al. 1993). EEG features of SRS have also been documented in rodent models of extended kindling. In amygdala-kindled rats, focal stage 0–2 motor seizures were associated with discharges in the kindled amygdala (Brandt et al. 2004) or ipsilateral dentate gyrus (DG) (Michael et al. 1998), and generalized stage 6–7 motor seizures were associated with discharges in bilateral amygdala (Pinel and Rovner 1978a), the kindled amygdala (Michael et al. 1998; Brandt et al. 2004), or ipsilateral DG (Michael et al. 1998). Generalized stage 3–5 motor seizures in association with kindled hippocampal discharges were observed from hippocampally kindled mice (Song et al. 2018a). Overall, however, EEG discharges from the kindled and unstimulated sites in individual rats or mice remain undetailed. It is unknown whether generalized motor seizures are initiated by concurrent bilateral discharges or discharges that originate from the kindled site and then spread to other brain regions as demonstrated in kindled monkeys (Wada et al. 1975; Wada and Osawa 1976) or cats (Hiyoshi et al. 1993).

Mouse models have been increasingly employed for epilepsy research largely due to advances in genetic/molecular manipulations that offer advantages to investigate the role of targeted molecular signaling in epileptogenesis. We attempt to establish a mouse model for future examinations of kindling-induced SRS in genetically/molecularly manipulated mice. Previous work of our laboratory has described protocols for extended hippocampal kindling and SRS monitoring in naïve C57 black mice (Bin et al. 2017), tested the effects of some of clinically used antiepileptic drugs on SRS (Song et al. 2018b), and examined performance of extended kindled mice in open filed and water maze tasks (Liu et al. 2019). Our present study is to continue characterization of this mouse model with a particular focus on electrographic features of SRS. Specifically, we recorded intracranial EEG from the kindled hippocampus and an unstimulated forebrain structure in individual mice to examine the temporal relation of corresponding regional discharges. The unstimulated structure was alternated in 5 groups of mice and targeted the hippocampus, parietal cortex, piriform cortex, entorhinal cortex, or dorsomedial thalamus. We also prepared brain slices from kindled and control mice to examine local circuitry excitability and susceptibility to induce epileptiform activity. Data from these in vivo and in vitro experiments suggest that epileptic activity involving a macroscopic network may generate concurrent discharges in forebrain areas and initiate SRS in hippocampally kindled mice.

## Materials and Methods

### Animals

Male C57 black mice (C57BL/6 N) were obtained from Charles River Laboratory (Saint-Constant, Quebec, Canada). Mice were housed in a local vivarium that was maintained at a temperature of 22–23°C and with a 12-h light on/off cycle (light-on starting at 6:00 AM). Mice were caged in group (up to 4 mice per cage) with food and water ad libitum. We chose to kindle mice (ages 11–13 months) to model new-onset temporal lobe epilepsy seen in adult and aging populations (Ferlazzo et al. 2016) while minimizing the health-related complications that are common in older mice (Flurkey et al. 2007). All experimentations conducted in this study were reviewed and approved by the Animal Care Committee of the University Health Network in accordance with the Guidelines of the Canadian Council on Animal Care.

### Electrode Implantation

Electrode construction and implantation were similarly done as per previous studies of our laboratory (Wu et al. 2008; Jeffrey et al. 2014; Bin et al. 2017; Stover et al. 2017; Song et al. 2018b). All electrodes were made of polyamide-insulated stainless steel wires (110 μm outer diameter; Plastics One). Surgeries were performed under isofluorane anesthesia. A stereotaxic frame with 2 micromanipulators was used for electrode placement. Implanted electrodes were secured onto the skull using a glue-based method (Wu et al. 2008).

Each mouse was implanted with 2 pairs of twisted-wire bipolar electrodes. One pair of electrodes was positioned to the hippocampal CA3 region for kindling stimulation and local recordings (bregma −2.5 mm, lateral 3.0 mm, and depth 3.0 mm; Franklin and Paxinos 1997), and another pair positioned to an ipsilateral or contralateral site. The latter was alternated in 5 groups of mice and targeted the contralateral hippocampal CA3, contralateral or ipsilateral parietal cortex (bregma −0.5 mm, lateral 2.0 mm, and depth 0.5 mm), ipsilateral piriform cortex (bregma 0.5 mm, lateral 3.0 mm, and depth 5.0 mm), contralateral dorsomedial thalamus (bregma −1.5 mm, lateral 0.5 mm, and depth 3.5 mm), or ipsilateral entorhinal cortex (bregma −3.5 mm, lateral 4.0 mm, and depth 5.0 mm). A reference electrode was positioned to a frontal area (bregma +1.5 mm, lateral 1.0 mm, and depth 0.5 mm). The putative tip locations of implanted electrodes were determined later in brain histological experiments if suitable (Supplementary Fig. 1).

We used the above implantation approach to assess regional EEG discharges while minimizing complications of multi-electrode implantations in the small mouse brain. Specifically, the rodent hippocampus has strong bilateral connections (Amaral and Witter 1998), which may allow faster spread of discharge signals from the kindled hippocampus to contralateral hippocampus relative to other brain structures, particularly the parietal cortex. The entorhinal and piriform circuitries are known to be susceptible to seizure activities and epileptogenesis (Vismer et al. 2015) and therefore may be prone to express spontaneous EEG discharges. Neurons of the dorsomedial thalamus have widespread connections with cortical and subcortical structures and have been shown to modulate seizure activities in other models (Bertram et al. 2001). Spontaneous EEG discharges have been observed from the dorsomedial thalamus of extended kindled cats (Hiyoshi et al. 1993). In the following text, the 5 different implantations were abbreviated as the hippo-hippo, hippo-cortex, hippo-piriform, hippo-thalamus, and hippo-entorhinal, respectively.

### Hippocampal Kindling

A train of stimuli at 60 Hz for 2 s was used for hippocampal kindling (Reddy and Rogawski 2010; Jeffrey et al. 2014; Bin et al. 2017; Stover et al. 2017; Song et al. 2018b). Constant current pulses with monophasic square waveforms, pulse duration of 0.5 ms and current intensities of 10–150 μA were generated by a Grass stimulator and delivered through a photoelectric isolation unit (model S88, Grass Medical Instruments). An ascending series was used to determine the threshold of evoked after-discharges (ADs) in individual mice. Kindling stimulation was conducted at 25% above the threshold value. We attempted to keep stimulation intensity constant throughout the extended kindling period. However, the initial stimulation intensity often became inconsistent in evoking ADs, which might be largely due to contaminations or fouling of the implanted electrodes. Due to this complication, stronger stimuli at 60–110% above the initial threshold value were used if needed.

Kindling stimuli were applied twice daily and ≥5 h apart (Pinel and Rovner 1978a, 1978b; Milgram et al. 1995; Mazarati et al. 1998; Sayin et al. 2003; Brandt et al. 2004; Bin et al. 2017). Each stimulation episode lasted for a few minutes while the mouse was placed in a glass container for EEG-video monitoring (Stover et al. 2017; Song et al. 2018b). Control mice were handled twice daily and ≥5 h apart for 60 days.

### E‌EG Recordings

Local differential recordings through the twisted-wire bipolar electrodes were used to monitor evoked responses and spontaneous EEG activities (Jeffrey et al. 2014; Bin et al. 2017; Stover et al. 2017; Song et al. 2018b). Monopolar EEG recordings were used only if the local differential recordings were unsuccessful. Discharges recorded by monopolar recordings were not used for analysis of regional discharges to avoid remote signal influences.

Evoked and spontaneous EEG signals were collected using 2-channel or 1-channel microelectrode AC amplifiers with extended head-stages (model 1800 or 3000, AM Systems). Evoked ADs of the stimulated hippocampus were captured using the model 3000 amplifier via TTL-gated switches between recording and stimulating modes. These amplifiers were set with an input frequency band of 0.1–1000 Hz and an amplification gain of 1000. A build-in notch filter at 60 ± 3 Hz was used in some experiments. Amplifier output signals were digitized at 5000 Hz (Digidata 1440A or 1550, Molecular Devices). Data acquisition, storage, and analyses were done using pClamp software (Version 10; Molecular Devices).

### Continuous EEG-Video Monitoring

Continuous 24-h EEG-video monitoring was done as previously described (Bin et al. 2017). Each mouse was placed in a modified cage with food and water ad libitum. A slip ring commutator was mounted atop the cage and connected to implanted electrodes of the mouse via flexible cables. A webcam (model C615, Logitech) was placed near the cage to capture animal motor behaviors. Video data were acquired at 20–25 frames per second. Dim lighting was used for webcam monitoring during the light-off period. A cursor auto-click program (Mini Mouse Macro program; http://www.turnssoft.com/mini-mouse-macro.html) was used to operate EEG and video recordings and save data every 2 h. EEG and video data were collected roughly 24 h daily and for up to 6 consecutive days per session. Supplementary Table 1 lists the cumulative time of 24-h EEG-video monitoring and SRS detected for individual mice.

**Figure 1:**
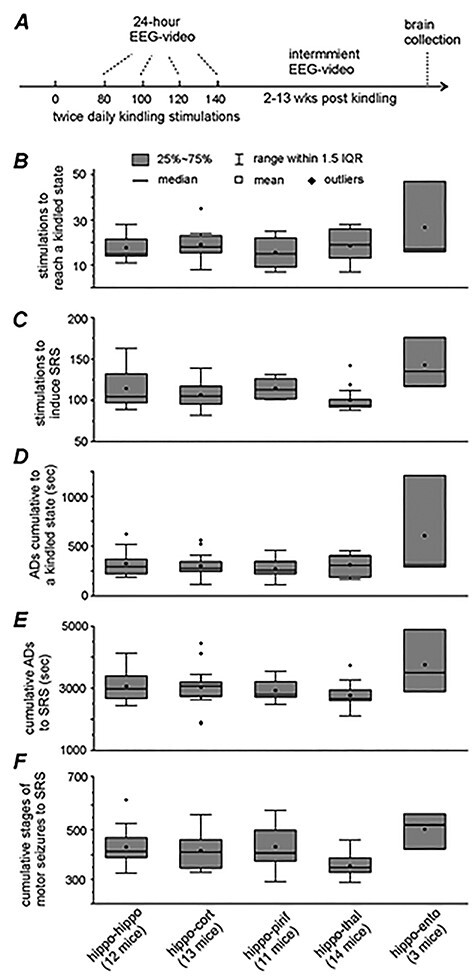
Kindling seizure progression. (*A*) An outline of experimental design. A total of 24-h EEG-video monitoring made after 80, 100, 120, and/or 140 stimuli. No stimulation applied if ≥2 SRS observed in the 24-h monitoring. Further EEG-video monitoring made intermittently for up to 3 months after termination of kindling. Brain tissues collected afterward for brain slice recordings or histological assessments. (*B*–*F*) Data from mice in the 5 implantation groups. (*B* and *C*) Numbers of stimuli needed to reach a kindled state and to induced SRS. (*D* and *E*) Cumulative durations of evoked ADs to the kindled state or SRS. (*F*) Cumulative stages of evoked motor seizures to SRS. These measures did not differ significantly among the hippo-hippo, hippo-cortex, hippo-piriform, and hippo-thalamus groups (*P* > 0.05, 1-way ANOVA following a Bonferroni post hoc test). Measures from the hippo-entorhinal group not included for group comparison due to a low sample size.

Individual mice underwent EEG-video monitoring for 24 h 1–2 weeks post electrode implantation and then after about 80, 100, 120, and 140 kindling stimulations. No further kindling stimulation was applied, if ≥2 SRS events were observed in the 24-h monitoring. Continuous 24-h EEG-video monitoring was performed intermittently for up to 3 months after termination of kindling stimulation (2–6 consecutive days per session, 2–3 sessions of 10–20 days apart) (Fig. 1*A*). Control mice experienced twice daily handling manipulations and underwent EEG-video monitoring for 24 h after 120 manipulations.

### Seizure Analysis

#### Electrographic Discharge Events

Evoked ADs and spontaneous discharges were recognized by repetitive spikes with simple and/or complex waveforms, amplitudes approximately 2 times the background signals, and durations of ≥10 s. Discharge events were inspected independently by 3 researchers (H.L., H.S., and L.Z.). Uncertain discharges (≤2% of SRS events in individual mice) were not included in the data presented below.

Hypersynchronous (HYP) and low-voltage fast (LVF) signals are 2 major onset patterns of EEG discharges recognized in patients with temporal lobe epilepsy and in relevant animal models (Bragin et al. 2005; Velasco et al. 2007; Lévesque et al. 2012; Avoli et al. 2016). The HYP onset was considered as a cluster of abruptly arising large spikes from the baseline. Spontaneous discharges that began with abrupt attenuation of the background activity were considered to have the LVF onset, although LVF associated rhythmic activity was not always evident to visual inspection. Specifically, the LVF-onset components were recognized by small amplitudes (≤65% of preceding signals in most cases) and variable lengths (0.5–5 s) prior to the appearance of repetitive incremental spikes. The magnitudes of LVF signals were measured from corresponding regional discharges without evident artifacts at onset. Original signals were treated with a band-pass filter (2–200 Hz, Bessel, 8-pole) to flatten DC-like changes associated with the LVF and to reduce high-frequency noises. Standard deviation (SD) values of the LVF-onset and preceding signals were obtained via signal analysis functions of pClamp. The SD of LVF signals were normalized as percentages of the preceding signals, and normalized measures from ≥20 discharge events were obtained from individual mice in the hippo-hippo, hippo-cortex, hippo-piriform, and hippo-thalamus groups (Supplementary Fig. 2).

#### Phase-Amplitude Coupling (PAC) and Wavelet Phase Coherence (WPC)

PAC strength was assessed using the algorithm by Tort et al. (2010) where phase and amplitude of different frequency bands were obtained via the complex wavelet Morlet wavelet transform using a mother wavelet with a central frequency of 0.8125 Hz and bandwidth of 5 Hz previously used on human EEG data (Grigorovsky et al. 2020). The phase and amplitude were computed from the real and imaginary wavelet coefficients using:

$$\begin{eqnarray*} \phi \left(\hat{t},{f}_L\right)=\arctan \frac{\mathit{\operatorname{Im}}\left\{W\left(\hat{t},{f}_L\right)\right\}}{\mathit{\operatorname{Re}}\left\{W\left(\hat{t},{f}_L\right)\right\}},\mathrm{and}\ A\left(\hat{t},{f}_H\right)\\ =\left|\mathit{\operatorname{Re}}\left\{W\left(\hat{t},{f}_H\right)\right\}+j\ \mathit{\operatorname{Im}}\left\{W\left(\hat{t},{f}_H\right)\right\}\right|,\mathrm{respectively}. \end{eqnarray*}$$

The low-frequency range used for the phase information was 1–30 Hz, and the high-frequency range used for the amplitude information was 32–512 Hz with increments on a logarithmic scale. The coupling strength is then computed as the normalized Kullback–Leibler distance of the amplitude distribution over the binned phases (20° bins) from a uniform distribution. The PAC strength was computed using a sliding window of 4 s to allow for a minimum of 4 cycles per frequency band.

PAC seizure dynamics were computed as the mean PAC strength using both spectral and temporal averaging. Peridischarge segments were chosen to be at least 8-s long to include at least 2 PAC windows and defined as preictal (8 s prior and up to electrographic onset); discharge or ictal onset segments were the first 8 s of the electrographic seizure; discharge or ictal segments were entire ictal trace excluding onset and offset segments; discharge or ictal offset segments were the last 8 s of the electrographic seizure; and postdischarge or postictal segments were 8 s following electrographic offset. PAC windows containing large movement artifacts were excluded from the analysis.

WPC was computed through the phase extraction of different frequency bands through the complex wavelet transform of EEG from each site (Cotic et al. 2015). The relative phase difference is obtained using $\Delta \phi (s,\tau )={\tan}^{-1}(\frac{W_1^{\ast}(s,\tau ){W}_2(s,\tau )-{W}_1(s,\tau ){W}_2^{\ast}(s,\tau )}{W_1(s,\tau ){W}_2(s,\tau )-{W}_1^{\ast}(s,\tau ){W}_2^{\ast}(s,\tau )})$ where ${W}^{\ast }$ is the complex conjugate, *s* is the scaling coefficient, and $\tau$ is the shift in time. Phase coherence is then defined as $\rho (s,\tau )=|\langle{e}^{j\Delta \phi (s,\tau )}\rangle |$, which ranges from zero to one, where one indicates a phase lock between the frequency bands of the 2 signals. WPC was applied to each wavelet central frequency from 0.25 to 512 Hz with increments on a logarithmic scale and window size proportional to 8 cycles of each frequency.

#### Motor Seizure Events

The Racine scale modified for mice (Racine 1972; Reddy and Rogawski 2010) was used to score evoked and spontaneous motor seizure activities. Briefly, stage 0—no response or behavioral arrest; stage 1—chewing or facial movement; stage 2—chewing, head nodding, and/or unilateral forelimb clonus; stage 3—bilateral forelimb clonus; stage 4—rear; stage 5—fall or a loss of righting reflex. In addition, both the evoked and spontaneous stage 3–5 motor seizures were often preceded by backward body movement and associated with tail erection, excessive salivation, and/or eye closure. Circular movements (≥3 turns) along the kindled or contralateral site (Pinel and Rovner 1978a) or brief fast runs were also noticeable before the spontaneous stage 3–5 motor seizures. The evoked and spontaneous motor seizures were assessed independently by several researchers (J.C., N.S., C.C., P.C., S.L., and Y.L.) through video reading (Jeffrey et al. 2014; Bin et al. 2017; Stover et al. 2017; Song et al. 2018b). The concordance rates for recognizing stage 3–5 seizures were ≥90% among these researchers.

#### SRS Detection

Combined EEG and video analyses were employed to detect SRS. EEG signals were first screened to detect spontaneous discharges. Detected discharge events were time-stamped, and corresponding video data were reviewed to score motor seizures. Discharge and motor seizure analyses were done separately as mentioned above. We used this approach for convenience in SRS detection as spontaneous discharges were clearly distinguishable from background signals in our EEG recordings, whereas scoring motor seizures through video reading was laborious and often complicated by mouse’s position in the cage and/or by surrounding bedding materials. Due to these complications in video reading, we did not analyze motor seizures by video reading alone. To be more stringent in SRS detection, SRS with decipherable discharges in 2 corresponding regional recordings and identifiable motor behaviors in video reading were presented below, and potential SRS events with decipherable EEG discharges from one recording site or without analyzable motor behaviors were not included in the present data presentation except where specified. In our EEG-video monitoring, EEG signals were recorded continuously, whereas video was captured at a rate of 20–25 frames per second and not synchronized with EEG recordings. Due to these limitations, we did not assess the temporal relation of EEG discharges and associated convulsive behaviors.

### Recordings in Brain Slices

Brain slice preparation, extracellular and whole-cell recordings, as well as data analyses were performed as previously described (Wu et al. 2005; El-Hayek et al. 2011; Moradi-Chameh et al. 2014; Song et al. 2018a). Briefly, each mouse was anesthetized by an intraperitoneal injection of sodium pentobarbital (100 mg/kg) and transcardially infused with cold, dissection-only artificial cerebrospinal fluid (ACSF) before decapitation. The brain was quickly dissected out and placed in ice-cold dissection only ACSF for 1–2 min for further cooling. Brain slices of 0.4 mm thickness were obtained using a vibratome (Leica VT1200) in ice-cold dissection only ACSF. Horizontal slices of the ventral part of the brain were collected to examine hippocampal, entorhinal, and piriform activities. After vibratome sectioning, slices were maintained in oxygenated (5%CO_2_–95%O_2_) standard ACSF for 1–6 h before recordings. The components of dissection only ACSF were (in mM): sucrose 280, KCl 3.5, CaCl_2_ 0.5, MgCl_2_ 6, HEPES 10, and D-glucose 10. The components of standard ACSF were (in mM): NaCl 125, KCl 3.5, NaH_2_PO_4_ 1.25, CaCl_2_ 2, MgSO_4_ 1.3, NaHCO_3_ 25, and D-glucose 10 (pH 7.35–7.4 when aerated with 5%CO_2_–95%O_2_). Epileptiform field potentials were induced using high-bicarbonate ACSF (Huberfeld et al. 2011), which contained (in mM) NaCl 71, KCl 6.5, NaHCO_3_ 80, NaH_2_PO_4_ 1.25, CaCl_2_ 1, MgSO_4_ 1, and D-glucose 10. When aerated with 5%CO_2_–95%O_2_, the pH of high-bicarbonate ACSF was 7.8–7.9.

All recordings were done in a submerged chamber and at a perfusate temperature of 35–36°C. Each slice was perfused with ACSF a high rate (~15 mL/min), and both the top and bottom surfaces of the slice were exposed to the perfused ACSF. Humidified gas of 95%O_2_–5%CO_2_ was passed over the perfusate to increase local oxygen tension. Previous works from our laboratory and other laboratories have shown that a fast both-side perfusion of rodent brain slices is important to maintain spontaneous population activities under submerged recording conditions (Wu et al. 2005; Hájos and Mody 2009).

A twisted-wire bipolar electrode made of polyimide-insulated stainless steel wires (outer diameter 110 μm) was used for local afferent stimulation. Constant current pulses (0.1 ms duration, at a near maximal intensity of 150 μA) were generated by a Grass stimulator (S88) and delivered through a photoelectric isolation unit. The stimulating electrode was placed near the cell body layer of the CA3 and DG area to elicit local responses.

Recording electrodes were made with thin-wall glass tubes (World Precision Instruments). Extracellular electrodes were filled with a solution containing 150 mM NaCl and 2 mM HEPES (pH 7.4; resistance of 1 ~ 2 MΩ). Patch electrodes were filled with an “intracellular” solution containing 140 mM potassium gluconate, 10 mM KCl, 2 mM HEPES, and 0.1 mM EGTA (pH 7.25 and resistance of 4–5 MΩ). A dual-channel amplifier (Multiclamp 700A or 700BA, Molecular Devices) was used to record extracellular filed potentials and intracellular signals. Data acquisition, storage, and analyses were done using a digitizer (Digidata 1400) and pClamp 10 software (Molecular Devices).

Slices that displayed stably evoked field potentials (≥0.2 or 0.5 mV for cortical or hippocampal responses) during baseline monitoring were included for data analysis. Synaptic field potentials were elicited every 20 s, and their peak amplitudes were measured from averages of 4–5 consecutive responses. Spontaneous CA3 sharp waves (SPWs) were recognized as rhythmic events with amplitudes approximately 2 times that of background signals, base durations of 20–200 ms, and incidences of 0.3–4 events/s. SPWs were visually inspected and measured from 1-min data segments in individual slices. Interictal spike events were recognized with amplitudes of ≥0.5 mV, base durations of 200–600 ms, and incidences of 2–5 events/10 s. An event detection function (threshold search method) of pClamp was used to automatically detect the interictal events. If needed, original data were treated with a band-pass filter (0.2–500 Hz, Bessel 8-pole) before event detection. Detected events were visually inspected and artifacts were excluded. SPW-related synaptic currents in CA3 pyramidal neurons were analyzed as previously described (Wu et al. 2005, 2006). About 15–20 events were collected at −60 or −40 mV for each neuron.

### Brain Histological Assessments

Brain histological sections were prepared using a protocol modified from previous studies of our laboratory (Jeffrey et al. 2014; Stover et al. 2017). Each mouse was anesthetized by sodium pentobarbital as described above and infused transcardiacally with standard ACSF and then with 10% neutral buffered formalin solution (Sigma-Aldrich). Removed brains were further fixed in a hypertonic formalin solution (with 20% sucrose) for ≥24 h. Brain coronal sections of 50 μm thickness were obtained using a Leica CM3050 research cryostat. Sections were mounted onto glass slides (Superfrost plusmicroscope slides, Fisher Scientific), dried at room temperature for ≥1 week, and then stained with cresyl violet (Sigma Aldrich). Images were obtained using a slide scanner (Aperio digital pathology slide scanner AT2, Leica; at ×20 magnification) and analyzed using ImageScope (Leica) or Image J software (National Institute of Health, USA).

### Statistical Analysis

Statistical tests were conducted using Sigmaplot (Systat Software Inc.) or Origin (One Roundhouse Plaza, Suite 303, Northampton, MA, USA) software. Data were presented as means and standard error of the mean (SEM) throughout the text and figures. Statistical significance was set at *P* < 0.05. For normally distributed data, group differences were assessed using a Student’s *t*-test or 1-way analysis of variance (ANOVA) followed by a Bonferroni post hoc test. When data were not distributed normally, a Mann–Whitney *U* test or a nonparametric ANOVA on rank (Kruskal–Wallis) followed by a post hoc test was used for group comparison. A Chi-square or Fisher exact test was used for comparing proportions.

## Results

### SRS Progression

We implanted 2 pairs of bipolar electrodes in each mouse. One pair of electrode was positioned in the hippocampal CA3 for kindling stimulation and local recording and another electrode positioned in an unstimulated structure. The latter was alternated in 5 groups of mice and targeted the contralateral hippocampal CA3, contralateral or ipsilateral parietal cortex, ipsilateral piriform cortex, contralateral dorsomedial thalamus, or ipsilateral entorhinal cortex. These different implantations were abbreviated as hippo-hippo, hippo-cortex, hippo-piriform, hippo-thalamus, and hippo-entorhinal, respectively.

Kindling seizure progression was assessed by the numbers of stimuli needed to reach a kindled state (3 consecutively evoked stage 5 motor seizures) or to induce SRS, cumulative evoked ADs, and motor seizures. For mice in the hippo-hippo, hippo-cortex, hippo-piriform, and hippo-thalamus groups (12, 13, 11, and 14 mice for each group), the numbers of kindling stimulations required to reach the kindled state were in a range of 15.5 ± 1.9 to 18.6 ± 2.0 stimuli (mean ± SEM hereinafter; Fig. 1*B*); cumulative durations of evoked ADs to the kindled state were ranged from 272.1 ± 27.8 to 325.3 ± 38.2 s (Fig. 1*D*). SRS were observed in the 4 groups of mice following 100.4 ± 4.0 to 114.8 ± 4.0 stimuli (Fig. 1*C*). Cumulative durations of evoked ADs to SRS were 2770.0 ± 110.3 to 3068.2 ± 145.7 s (Fig. 1*E*); cumulative stages of evoked motor seizures to SRS were 356.3 ± 12.7 to 432.5 ± 25.9 (Fig. 1*F*). There was no significant group difference among these measures (1-way ANOVA), suggesting that SRS progression is not substantially influenced by electrode implantations in different unstimulated structures. Measures were similarly obtained from 3 mice in the hippo-entorhinal group but not included for group comparison due to small sample size.

Hippocampal kindling was terminated in 17 other mice after 23–96 stimuli due to loss/malfunction of implanted electrodes or health-related complications. Aberrant hippocampal spikes with peak amplitudes of ≥2 times of background signals and intermittent incidences of 1–5 events/10 s, but not SRS, were observed in 2/17 mice while being monitored after 80 stimulations. Control mice (*n* = 10) received electrode implantations in hippocampal and piriform/cortical areas and experienced twice daily handling manipulations. Neither seizures nor aberrant hippocampal spikes were observed from these control mice while being monitored after 120 handling manipulations. Together these observations suggest that SRS result from a sufficient cumulation of evoked seizure activity and that the chronic handling manipulation and intracranial electrode implantation per se do not induce seizure activity.

### Motor Seizure Expression of SRS

Spontaneous motor seizures were assessed using the Racine scale modified for mice (Racine 1972; Reddy and Rogawski 2010; Reddy and Mohan 2011) because they were like evoked motor seizures. Briefly, stage 0, 1, and 2 motor seizures were recognized by behavioral arrest, chewing/facial movement, and head nodding/unilateral forelimb clonus; stage 3, 4, and 5 motor seizures were recognized by bilateral forelimb clonus, rearing, and falling, respectively (Supplementary Videos 1–3). A total of 3244 SRS events with identifiable motor behaviors were captured from 45 mice. These included 310 SRS events collected from 5 mice in the hippo-hippo group, 498 events from 13 mice in the hippo-cortex group, 269 events from 11 mice in the hippo-piriform group, 2113 events from 14 mice in the hippo-thalamus group, and 54 events from 3 mice in the hippo-entorhinal group, respectively.

SRS with expression of stage 3, 4, or 5 motor seizures were predominantly observed from individual mice in the hippo-hippo, hippo-cortex, hippo-piriform, and hippo-entorhinal groups. Collectively, SRS with expression of stage 3–5 motor seizures accounted for 85.2–100% of total SRS events observed from the 4 groups of mice. SRS with expression of stage 0, 1, or 2 motor seizures were more frequently observed from individual mice of the hippo-thalamus group. In addition, SRS with consecutive expression of stage 0, 1, or 2 motor seizures were observed from 4 mice in this group (interevent intervals of 3–15 min and 3–17 events/episode). Collectively, SRS with expression of stage 0–2 motor seizures made up 57.1% of the total SRS observed from mice of the hippo-thalamus group, which were significantly greater than those observed from mice in the hippo-hippo, hippo-piriform, or hippo-cortex group (Chi-square test, *P* < 0.001). We therefore suggest that SRS with expression of stage 3–5 motor seizures are prominent in hippocampally kindled mice and that the severity of motor seizures may be influenced by chronic implantation of thalamic electrodes in some mice (see Discussion).

### E‌EG Features of SRS

#### Coexpressed Discharges in Kindled Hippocampal and Corresponding Unstimulated Areas

Spontaneous discharges were recognized by repetitive spike waveforms with amplitudes approximately 2 times that of background signals and durations of ≥10 s. Most discharges began with LVF signals (see below), which were followed by incremental rhythmic spikes and then sustained large-amplitude spikes with simple or complex waveforms. Discharge termination in most cases featured a sudden cessation of spike activity and a subsequent component of signal suppression lasting several seconds. Discharge durations were determined by the time between the LVF onset and the spike cessation.

**Figure 2:**
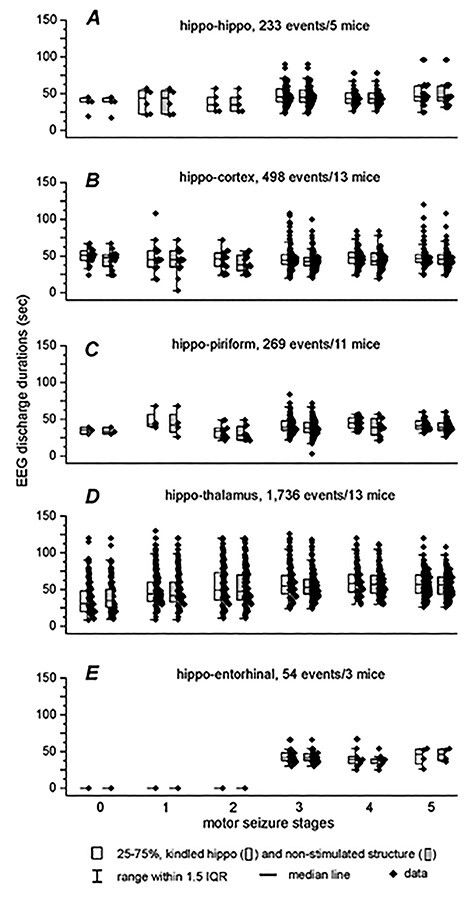
Relations of EEG discharge durations and motor seizure stages. Discharges and associated motor seizures were assessed for individual SRS events. (*A–E*) Measures from mice in the 5 implantation groups were presented. Numbers of SRS events and mice examined in each group were indicated. There was no significant difference between durations of stimulated and unstimulated sites at each stage of motor seizures (Student’s *t* test or Mann–Whitney *U* test). Regional discharge durations corresponding to stage 1–5 motor seizures were not significantly different in each group (nonparametric ANOVA on rank test). There was no strong correlation between regional discharge durations and motor seizure stages (linear regression analysis, *R*^2^ = 0.001–0.022).

A total of 2790 SRS events with decipherable EEG signals in both corresponding regional recordings and identifiable motor behaviors in video were collected from the 5 groups of mice. These included 233 events from 5 mice in the hippo-hippo group, 498 events from 13 mice in the hippo-cortex group, 269 events from 11 mice in the hippo-piriform group, 1736 events from 13 mice in the hippo-thalamus group, and 54 events from 3 mice in the hippo-entorhinal groups, respectively (Fig. 2). Measures from some events during the abovementioned consecutive stage 0–2 motor seizures were not included in Figure 2. On these occasions, discharges were evident in kindled hippocampal recordings but barely recognizable in corresponding thalamic recordings (Supplementary Fig. 3).

Coexpressed discharges in the kindled hippocampus and corresponding unstimulated structure were observed in all 2790 SRS events captured. Durations of corresponding regional discharges were not significantly different in the 5 groups of mice, irrespective of stages of associated motor seizures (Student’s *t*-test or Mann–Whitney *U* test; Fig. 2*A*–*E*). There was no significant difference among discharge durations sorted according to motor seizure stages in each group (nonparametric ANOVA on rank test). Linear regression analysis revealed no strong correlation between durations of regional discharges and the stages of motor seizures in each of the 5 groups of mice (*R*^2^ = 0.01–0.02). Similar coexpression of corresponding regional discharges was also observed when associated motor seizures were unanalyzable due to complications in video monitoring (data not shown). However, some thalamic discharges were unappreciable during the abovementioned consecutive stage 0–2 motor seizures (Supplementary Fig. 3*B,C*).

#### Corresponding Regional Discharges Displayed Concurrent LVF Onsets

**Figure 3:**
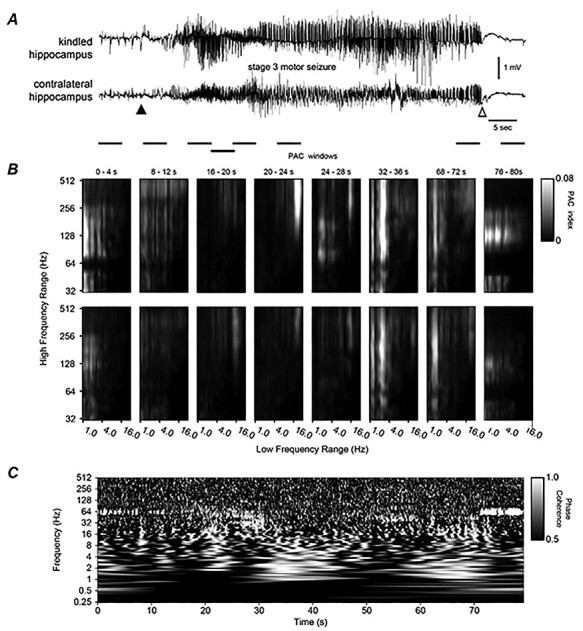
Bilateral hippocampal activities and corresponding PAC, WPC analyses. (*A*) Original EEG signals collected in a frequency band of 0.1–1000 Hz were illustrated. Putative discharge onset or termination denoted by a filled and open arrow. Discharges were associated with a stage 3 motor seizure. (*B*) PAC of kindled (top) and unstimulated (bottom) hippocampal activities. Eight sequential windows with indicated times matched horizontal bars below traces in (*A*). The low-frequency range used for the phase information was 1–30 Hz and the high-frequency range used for the amplitude information was 32–512 Hz, with increments on a logarithmic scale. Note that PAC in window 4 between 20–25 Hz (*x*) and 128–512 Hz (*y*) signals and in window 6 between 2–3 and 64–512 Hz signals were stronger in the top than in bottom panels. (*C*) WPC plot for the corresponding regional signals in (*A*). WPC was applied to each wavelet central frequency from 0.25 to 512 Hz with increments on a logarithmic scale and window size proportional to 8 cycles of each frequency. Scale 1 indicated a phase lock. Note phase-locked/near phase-lock discharge signals appearing around the 20–32 s time stamps and in a frequency range of 20–80 Hz as well as around the 30–40 and 56–70 s time stamps and in a frequency range of 1–16 Hz. Also, note these coherent signals were accompanied with dissimilar regional PAC in windows 3–7.

**Figure 4:**
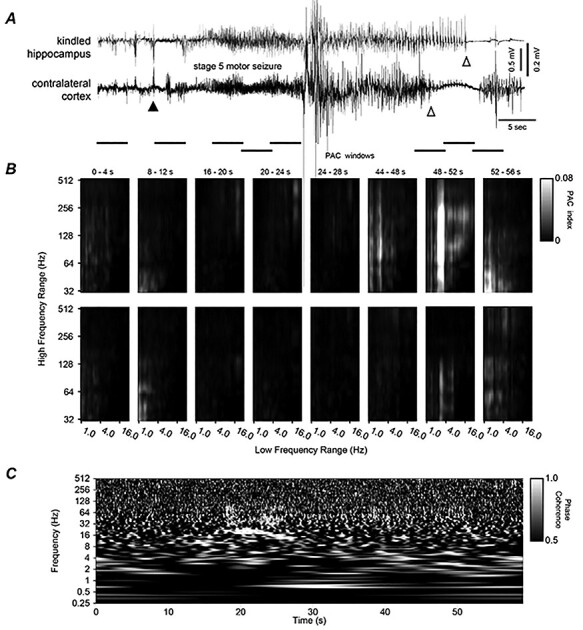
Hippocampal–cortical discharges, corresponding PAC, and WPC analyses. (*A*) Original EEG signals similarly illustrated as in Figure 3. Discharges were associated with a stage 5 motor seizure (Supplementary Video 1). Note large artifacts in middle discharges and different termination times of regional discharges. (*B* and *C*) PAC and WPC plots similarly arranged as in Figure 3. Note in (*B*) and in windows 6–7 there were stronger PAC between 2–3 and 32–256 Hz signals in the top (hippocampal) than bottom (cortical) panels. Note in (*C*) phase-locked or near phase-locked signals appearing around in the 18–28 s time stamps and in frequency ranges of 10–20 as well as 30–80 Hz.

**Figure 5:**
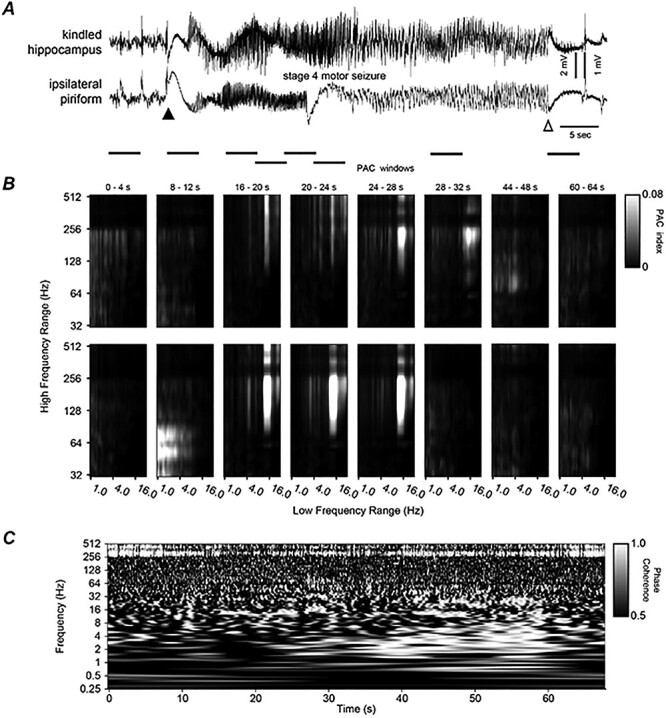
Hippocampal–piriform discharges, corresponding PAC, and WPC analyses. (*A*) Original EEG signals similarly illustrated as in Figure 3. Discharges were associated with a stage 4 motor seizure (Supplementary Video 2). (*B* and *C*) PAC and WPC plots similarly arranged as in Figure 3. Note in (*B*) and in windows 3–5 there were stronger PAC between roughly 12–20 and 64–256 Hz signals in the bottom (piriform) than in top (hippocampus) panels. Note in (*C*) phase-lock or near phase-locked discharge signals appearing around in the 15–30 s time stamps and in at frequencies around 16 Hz as well as in the 35–60 s time stamps and in a lower (1–5 Hz) and a higher (15–25 Hz) frequency range. These coherent signals were accompanied with dissimilar regional PAC in windows 3–6.

**Figure 6:**
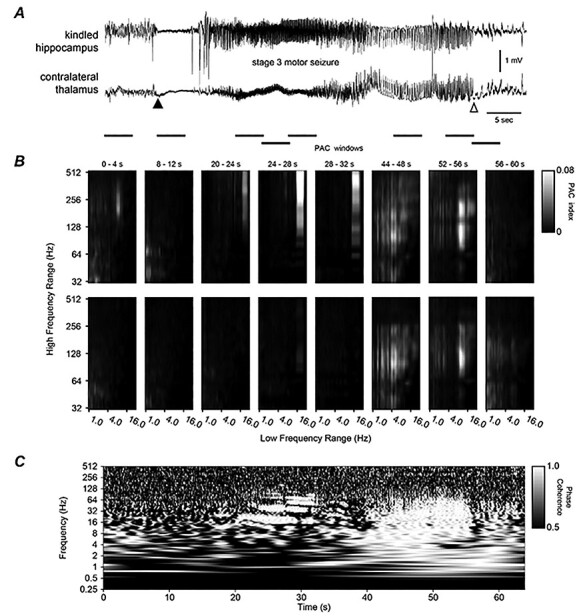
Hippocampal–thalamic discharges, corresponding PAC, and WPC plots. (*A*) Original EEG signals similarly illustrated as in Figure 3. Discharges were associated with a stage 3 motor seizure (Supplementary video 3). (*B* and *C*) PAC and WPC plots similarly arranged as in Figure 3. Note in (*B*) and in windows 4–5 there were stronger PAC between 20–25 and 128–512 Hz signals in top (hippocampus) than low (thalamus) panels. Note in (*C*) phase-locked or near phase-locked discharge signals appearing around in the 18–32 s time stamps and in a frequency range of 16–80 Hz as well as in the 36–56 s time stamps and in a frequency range of 4–80 Hz. The later coherent signals were accompanied with seemingly similar regional PAC in windows 6–7.

Most discharges began with LVF signals, which were considered as components that displayed small amplitudes (≤65% of preceding signals in most cases; see Materials and Methods; Supplementary Fig. 2) and durations of 0.5–5 s prior to appearance of repetitive incremental spikes. The LVF signals often appeared right after a spike with simple or complex waveform (Figs 3*A*–5*A*, Supplementary Figs 3*A*–7*A*) and/or overlaid onto a slow baseline shift (Fig. 5*A*; Supplementary Figs 2*A*, 7*A*). The latter might represent an altered DC signal due to the input frequency range (0.1–1000 Hz) of the amplifiers used in our recordings (see Materials and Methods). It was difficult to systematically define the starts of regional LVF signals because of the variable signals immediately preceding the LVF onset. We therefore considered the LVF signals start from the immediately preceding spikes (Figs 3*A*–5*A*; Supplementary Figs 3*A*–7*A*) or from time points at which signal amplitudes were abruptly and markedly attenuated from preceding signals (Fig. 6*A*, Supplementary Fig. 8*A*).

The LVF onsets of corresponding regional discharges consistently showed a concurrent relation irrespective of the unstimulated structure targeted. For example, LVF signals with immediately preceding spikes were observed from bilateral hippocampal discharges (Fig. 3*A*; Supplementary Fig. 5*A*) and hippocampal–cortical discharges (Fig. 4*A*; Supplementary Fig. 6*A*), and these spikes occurred nearly synchronously in bilateral hippocampal and hippocampal–cortical recording. The relation of corresponding regional LVF onsets was independent of discharge durations and associated motor seizures. As shown in Supplementary Figures 5*A*, 7*A*, 8*A*, discharges associated with stage 1 or 2 motor seizures were much shorter than those associated with stage 3 or 4 motor seizures (Figs 3*A*, 5*A*, 6*A*), but their LVF onsets were highly comparable in waveform and concurrence.

In contrast to the consistently observed LVF onset, 4 hippocampal discharges were considered to begin with HYP signals, which featured a cluster of abruptly arising large spikes. These HYP discharges were observed from one mouse within 18 h after termination of hippocampal kindling (Supplementary Fig. 10*A*), whereas all subsequent discharges observed from same mouse were found to begin with the LVF signals (Supplementary Fig. 10*B*). Additionally, some hippocampal discharges began with repetitive incremental spikes, and these discharges were observed only during the above-mentioned consecutive stage 0–2 motor seizures (Supplementary Fig. 3*B*,*C*). Together, these observations suggest that the LVF signals are a dominant onset pattern of spontaneous EEG discharges in hippocampally kindled mice.

#### Corresponding Regional Discharges Displayed Different Waveforms

**Figure 7:**
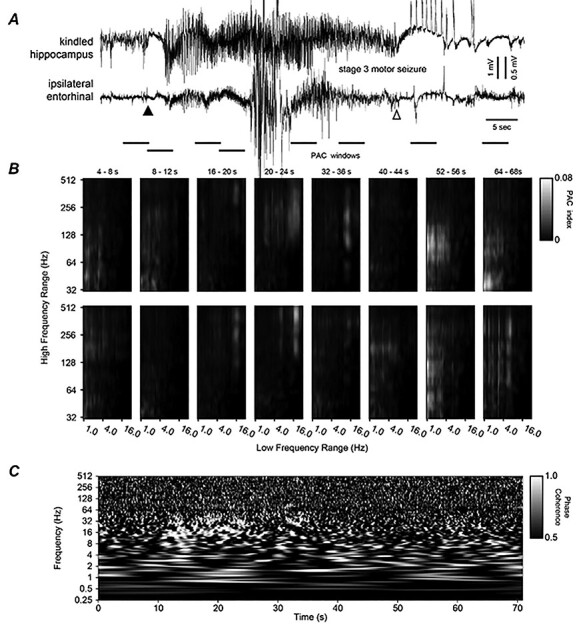
Hippocampal–entorhinal discharges, corresponding PAC, and WPC plots. (*A*) Original EEG signals similarly illustrated as in Figure 3. Discharges were associated with a stage 3 motor seizure. Note large artifacts in middle discharges and large amplitude spikes following hippocampal discharge. (*B* and *C*) PAC and WPC plots similarly arranged as in Figure 3. Note in (*B*) and in windows 3–6 there were weak but different regional (hippocampal vs. entorhinal) PAC. Note in (*C*) phase-locked or near phase-locked discharge signals appearing around in the 11–24 s time stamps and in a frequency range of roughly 10–32 Hz.

We conducted local differential recordings through twisted wire bipolar electrodes to sample “local” signals while attenuating the influences of remote signals. Region-specific activities were observed from the kindled hippocampus and corresponding unstimulated ipsilateral/contralateral structure. Specifically, discharges simultaneously recorded from the 2 corresponding areas were different in waveform (Figs 3*A*–7*A*) and/or termination times (Fig. 4*A*; Supplementary Fig. 6). In addition, large amplitudes spikes were observed from the kindled hippocampus but not corresponding unstimulated cortical, piriform, entorhinal, or thalamic area (Fig. 7*A*, Supplementary Fig. 4*A*,*B*). These spikes appeared several seconds after hippocampal discharges and displayed variable spike rates (3–5 spikes/s) and durations (19.7 ± 1.3 s/episode, 79 events from 12 mice). Small amplitude hippocampal spikes were also observed before or after discharges (21 ± 2.1 s/episode, 19 events/5 mice, Supplementary Fig. 5*A*). We speculate that the hippocampal spikes might largely represent “local” hyperexcitability of the kindled hippocampal circuitry. Furthermore, in recordings made from some mice in the hippo-thalamus group and during the abovementioned consecutive stage 0–2 motor seizures, discharges were evident in the kindling hippocampal area but unappreciable in the corresponding thalamic area (Supplementary Fig. 3*B,C*).

#### PAC and WPC analyses

**Figure 8:**
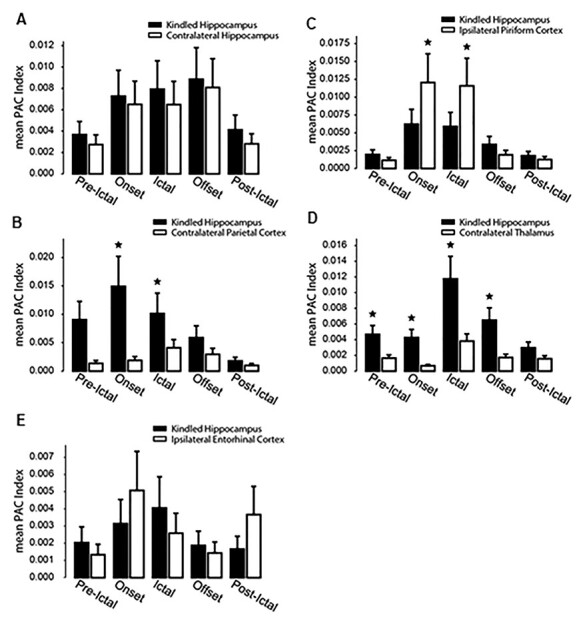
Summaries of regional PAC. (*A–E*) corresponding regional discharges from mice in the 5 implantation groups were analyzed (1–2 discharge events per mouse and 3–5 mice per implantation group). Mean PAC indexes were computed using both spectral and temporal averaging. Discharges were abbreviated as ictal in *x*-axis. Peri-ictal segments were chosen to be at least 8 s long to include at least 2 PAC windows. Onset sections were the first 8 s of the electrographic discharge onset. Ictal sections included entire discharges excluding onset and offset segments. Offset sections were the last 8 s of the electrographic discharges. Postictal sections were 8 s following electrographic discharge termination. PAC windows containing large movement artifacts were excluded from the analysis. ^*^, Kindled hippocampus versus corresponding unstimulated structure, *P* < 0.05, Mann–Whitney *U* test. Note in (*B* and *D*) significantly stronger PAC in the kindled hippocampus than in corresponding unstimulated cortex or thalamus. Also note in (*C*) significantly weaker PAC in the kindled hippocampus relative to corresponding unstimulated piriform cortex.

PAC analysis was performed for a set of 28 corresponding regional discharge (1–2 events per mouse and 3–5 mice per group for the 5 implantation groups). PAC strengths were assessed in 8 sliding 4-s time windows (Weiss et al. 2013; Zhang et al. 2017; Grigorovsky et al. 2020) that matched pre- and postdischarge signals as well as the onset, middle, and offset epochs of regional discharges (Grigorovsky et al. 2020). The slow oscillation used for the phase information was 1–30 Hz and the fast oscillation used for the amplitude information was 32–512 Hz. Regional discharges appeared to show a general trend in PAC features: weak PAC mainly between the phase of 1–4 Hz and the amplitude of 32 up to 256 Hz signals were noticeable for discharge onset epochs; stronger PAC between the phase of 8–12 or 16–20 Hz and the amplitude of 64 up to 512 signals were evident for middle and later discharge epochs; PAC for postdischarge signals were like those for discharge onset epochs (panel B of Figs 3–7 and Supplementary Figs 5–9). Overall, the patterns and strengths of PAC were distinct between corresponding discharge events analyzed. When data from each implantation group were pooled together, mean PAC indexes for the onset and middle parts of discharges were significantly greater for the kindled hippocampus than for the cortex or thalamus (Fig. 8*B*,*D*) but weaker for the hippocampus relative to the piriform cortex (Fig. 8*C*).

WPC analysis was performed for the 28 corresponding discharge events mentioned above. WPC was computed through the phase extraction of different frequency bands from 0.25 to 512 Hz. Phase differences of corresponding 2 signals were scaled in a range of 0–1, with 1 indicating a phase-lock. In general, the onsets of corresponding regional discharges, irrespective of the unstimulated structures targeted, did not show an evident increase in coherent signals as compared with predischarge activities. Phase-locked or near phase-locked signals were evident for the early, middle, and/or later parts of corresponding regional discharge. These coherent signals varied in length and expressed in relatively narrow frequency bands from approximately 10 Hz up to 80 Hz in most cases (panel C of Figs 3–5, 7 and Supplementary Figs 5–9). Coherent discharge signals with more persistent expression in a wider frequency range of 10–100 Hz were occasionally observed (Fig. 6*C*). In addition, coherent discharge signals with more uniform expression in a low-frequency range of 1–5 Hz were also noticeable in some cases (Figs 3*C*, 5*C*, 6*C*; Supplementary Fig. 9*C*). Overall, the occurrence of these coherent discharge signals was accompanied with dissimilar regional PAC (panel B of Figs 3–7 and Supplementary Figs 5–9). Taking together the electrographic observations and the results of PAC and WPC analyses, we postulate that the concurrent regional discharges observed in our present experiments cannot be entirely attributable to remote volume conducted signals (see Discussion).

### Baseline Activity and Induced Epileptiform Field Potentials Observed From Brain Slices

The above observations suggest that corresponding regional EEG discharges with concurrent LV onsets are a main electrographic feature of SRS in hippocampally kindled mice. However, these observations were limited as spontaneous discharges were monitored from only 2 sites in each mouse and it was difficult to assess the temporal relation of corresponding regional LVF onsets. If spontaneous discharges were originated primarily from the kindled hippocampus and then spread to other brain structures for generation, one would anticipate that the kindled hippocampal CA3 circuitry may be more susceptible than other unstimulated forebrain circuitries for genesis of epileptiform activities when examined in vitro. We therefore conducted brain slice experiments to explore this. Horizontal brain slices (0.4 mm thick) encompassing ventral hippocampal and entorhinal areas or ventral–medial piriform area were prepared from extended kindled mice with SRS. Slices similarly prepared from mice after chronic handling manipulations or electrode implantation alone served as controls. Extracellular and whole-cell patch recordings were used to monitor local field potentials and single-cell activities. Activities of the hippocampal CA3 and DG areas were examined in slices ipsilateral to the kindling site, and piriform and entorhinal activities were monitored from slices ipsilateral or contralateral to the kindling site. All recordings were done in a submerged chamber and at a perfusate temperature of 35–36°C.

#### “Baseline” CA3 and DG Activities

**Figure 9:**
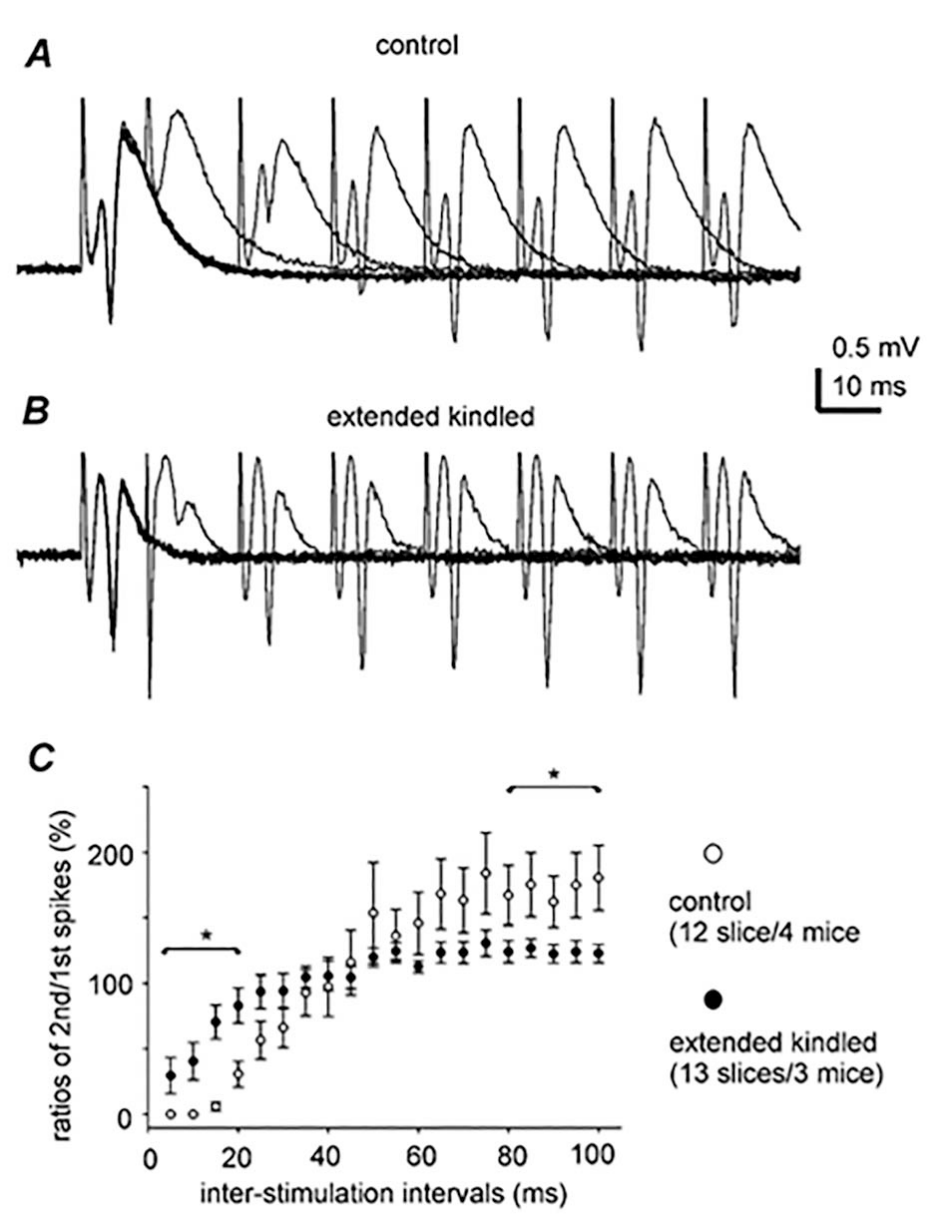
Effects of paired stimulations on DG population spikes. (*A* and *B*) Representative traces collected from slices of a control mouse (*A*) and a kindled mouse (*B*). Population spikes were evoked by paired stimuli with interstimulus intervals of 10–80 ms. Traces were superimposed for illustration purpose. Note that in response to paired stimuli with an interval of 10 ms, the second stimulus evoked a small spike in (*B*) (kindled) but not in (*A*) (control). (*C*) Data collected from 12 slices of 4 control mice and from 13 slices of 5 kindled mice. *y-*axis, the amplitude ratios (%, mean ± SE) of the second versus the second spikes. *x-*axis, interstimulus intervals. ^*^Kindled versus control, Student’s *t*-test, *P* < 0.05. Note that spike inhibition or enhancement by paired stimuli at intervals of 5–20 or 80–100 ms were attenuated in slices of kindled mice.

Slices perfused with standard ACSF were used to examine whether CA3 and DG “baseline” activities are altered by extended hippocampal kindling. CA3 or DG population spikes were evoked by local afferent stimulation at near maximal intensity and recorded from the cell body layer. The peak amplitudes of CA3 and DG population spikes were comparable in slices of kindled and control mice (Table 1), but effects of paired stimuli on DG spikes were different between the 2 groups of slices. Spike inhibition by paired stimuli 5–20 ms apart and spike enhancement by paired stimuli at intervals of 80–100 ms were significantly attenuated in slices of kindled mice as compared with slices of control mice (Fig. 9). In CA3 pyramidal neurons monitored by whole-cell current-clamp recordings, resting membrane potentials, action potential peak amplitudes, and half-widths were not significantly different between neurons of kindled and control mice, but input resistance measures were lower in kindled CA3 neurons (Table 1). The latter observation might be partly due to increased synaptic activities hence decreased membrane resistance described below.

**Table 1:** Extracellular and intracellular measurements in hippocampal slices

	Control mice	Kindled mice with SRS
Extracellular measures		
CA3 population spikes (mV)	2.03 ± 0.23 (50 slices/10 mice)	1.68 ± 0.12 (36 slices/6 mice)
DG population spikes (mV)	2.10 ± 0.12 (12 slices/4 mice)	1.90 ± 0.05 (13 slices/3 mice)
Measures from CA3 pyramidal neurons		
Resting membrane potentials (mV)	−60.5 ± 0.7	−56.2 ± 3.6
Input resistance (MΩ)	195.0 ± 10.6	120.9 ± 6.5^*^
Action potential peak amplitudes mV)	111.3 ± 1.5	103.0 ± 2.0
Action potential half-width (ms)	1.02 ± 0.04	1.06 ± 0.04
Action potential voltage threshold (mV)	−42.8 ± 0.9	−44.4 ± 0.7
	35 cells/10 mice	30 cells/12 mice

^*^Note: Control versus extended kindled, Student’s *t*-test, *P* = 0.025.

**Figure 10:**
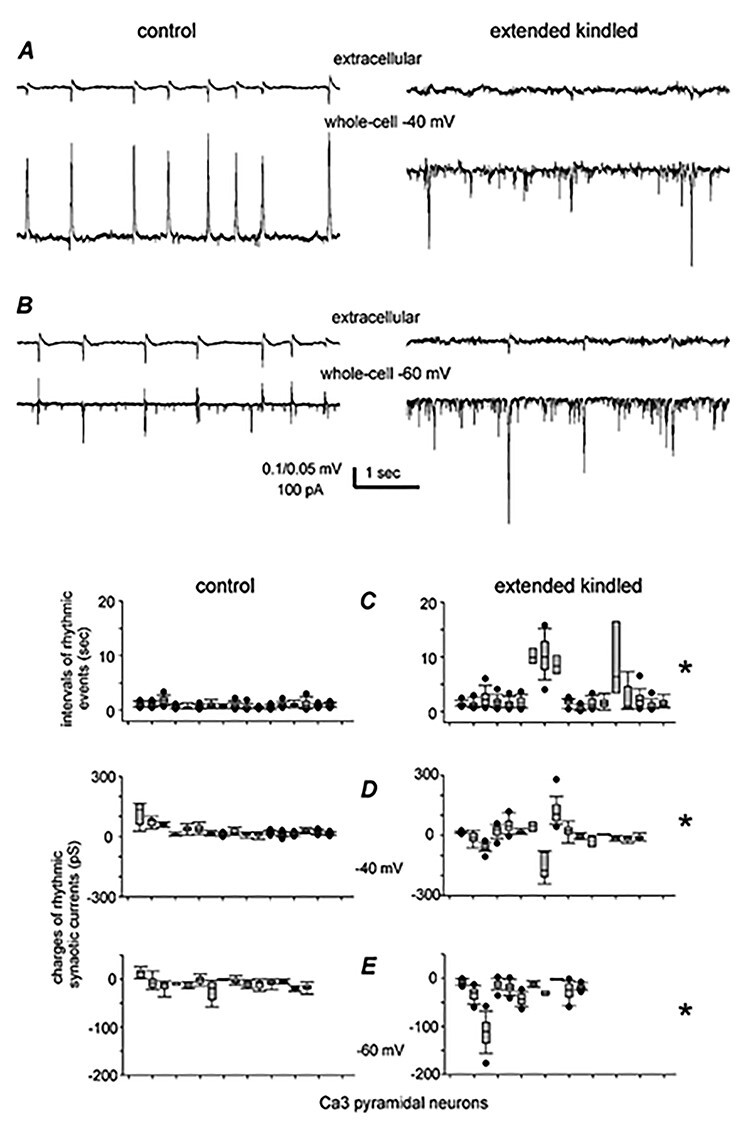
CA3 in vitro SPWs and related synaptic currents. (*A* and *B*) 2 CA3 pyramidal neurons voltage clamped at −40 (*A*) and − 60 mV (*B*) together with local extracellular recordings. Note smaller SPW-related outward (upward) components at −40 mV and larger inward (downward) components at −60 mV in kindled than in control neuron. (*C*–*E*) Data collected from 18 neurons of 4 control mice and from 17 neurons of 5 kindled mice. ^*^, Kindled versus controls, median values comparisons, *P* ≤ 0.05, Student’s *t*-test, or Mann–Whitney *U* test. Note in (*C*) less frequent (with longer intervals) CA3 SPWs in slices of kindled mice. Also, note in (*D*) that synaptic conductance measured at −40 mV were outward dominant in control neurons but mixed inward–outward in kindled neurons. In (*E*), synaptic conductance measures at −60 mV were mixed inward and outward components in control neurons but robust inward components in kindled neurons.

Rodent hippocampal slices can exhibit spontaneous field potentials or in vitro sharp waves (SPWs; Karlócai et al. 2014; Buzsáki 2015). Previous work from our laboratory has suggested that in vitro SPWs of the mouse hippocampus are originated from the CA3 area and generated by local network activities involving both glutamatergic and GABAergic synapses (Wu et al. 2005, 2006). We therefore examined whether CA3 in vitro SPWs are altered in extended kindled mice with SRS. Monitored by extracellular recordings in 18 slices from kindled mice and 17 slices of control mice, CA3 SPWs were smaller and less frequent in kindled relative to control mice (Fig. 10*A*–*C*). SPW-related synaptic currents in CA3 pyramidal neurons were also altered in kindled mice. When voltage clamped at −40 mV, SPW-related inhibitory postsynaptic currents (IPSCs) were barely detectable in neurons of kindled mice but robust in neurons of control mice (Fig. 10*A*). When held at −60 mV, CA3 neurons of kindled mice displayed large excitatory postsynaptic currents (EPSCs), whereas neurons of control mice showed mixed EPSCs/IPSCs in correlation with local SPWs (Fig. 10*B*). Overall, outward (inhibitory) synaptic conductance at −40 mV was smaller, whereas inward (excitatory) conductance at −60 mV was greater in kindled than in control CA3 neurons (18 or 17 neurons from 4 or 5 mice in each group, Fig. 10*D*,*E*). However, the reversal potentials of evoked IPSCs, assessed in the presence of a general glutamate receptor antagonist kynurenic acid (2.5 mM), were not significantly different between kindled and control CA3 pyramidal neurons (−70.3 ± 2.1 mV vs. −72.4 ± 1.6 mV, *n* = 6 or 7 neurons, Supplementary Fig. 11). Epileptiform field potentials with multiple spikes and prolonged waveforms, spontaneously occurring or induced by single or high-frequency (80 Hz for 1 s) stimulation, were not observed from the CA3 and DG as well as from piriform and entorhinal areas in all slices examined (66 from 12 kindled mice and 82 slices from 16 control mice).

#### Induced Epileptiform Field Potentials

The above observations suggest that the CA3 and DG circuitries may be altered toward hyperexcitability in slices of extended kindled mice, but such alterations do not cause population epileptiform activity in slices perfused with standard ACSF. We then perfused slices with high-bicarbonate ACSF (alkaline pH 7.8–7.9; see Materials and Methods) to induce epileptiform field potentials and to explore whether susceptibility for induced epileptiform activity is altered in slices of kindled mice. We used this approach as similar high-bicarbonate ACSF reliably induced ictal-like discharges in cortical slices prepared from surgical specimens of epilepsy patients (Huberfeld et al. 2011). Additionally, in our pilot experiments, high-bicarbonate ACSF was more consistent than low-Mg^2+^ ACSF (0.5 mM), high-K^+^ ACSF (8–10 mM) or ACSF containing 4-aminopyramine (100 μM) to induce ictal discharges in slices of kindled mice.

**Figure 11:**
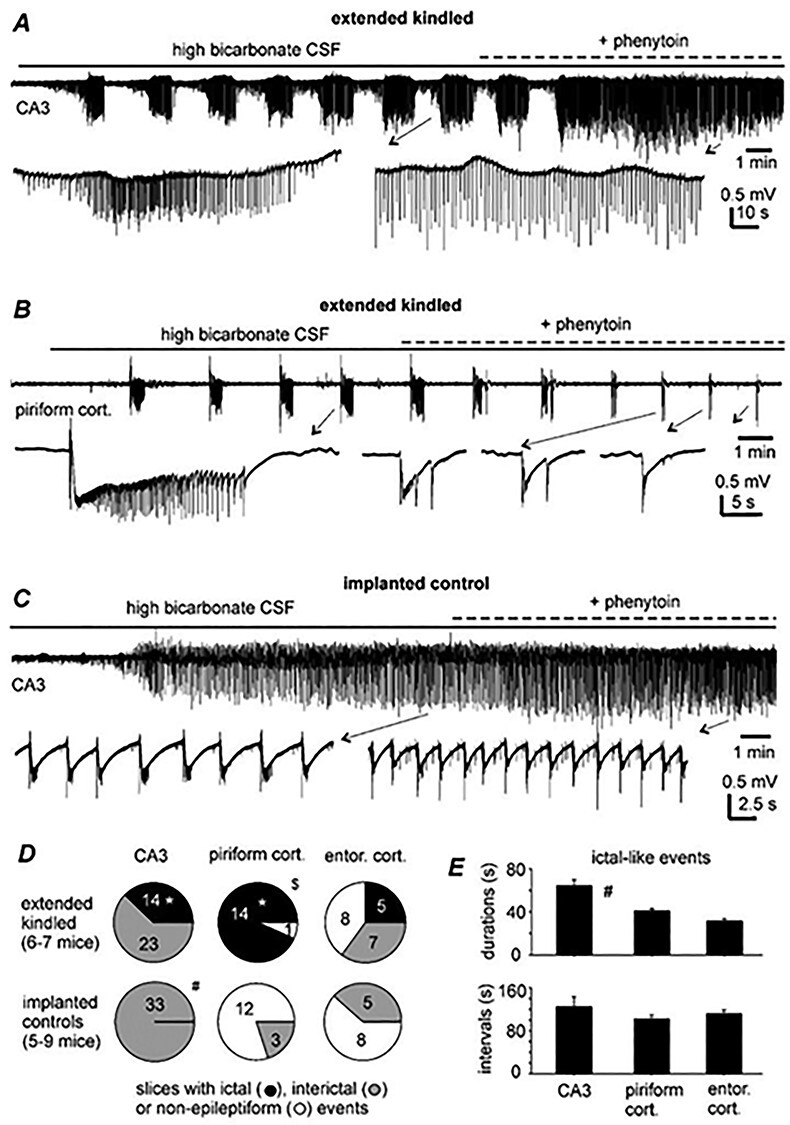
Epileptiform field potentials induced by high-bicarbonate ACSF. (*A*–*C*) Extracellular traces collected from slices of 2 kindled mice and a control mouse. Top, continuous traces illustrated after treatment with a band-pass filter (2–500 Hz). The application times of phenytoin (100 μM) indicated above traces. Arrowed events illustrated in a wide frequency range (0–1000 Hz). Note in (*A* and *B*) phenytoin suppressed ictal discharges but not interictal spikes. Also note in (*C*) interictal spikes persisted following phenytoin application. (*D*) Proportions of slices with or without induced epileptiform field potentials. ^*^, Kindled versus control; $, piriform versus CA3 or entorhinal area; #, CA3 versus piriform or entorhinal area; Chi-square or Fisher’s exact test, *P* < 0.05. (*E*) Discharges measured from the CA3, piriform, and entorhinal areas (5–14 events/area). #CA3 versus piriform or entorhinal area, 1-way ANOVA, *P* < 0.05.

Two main types of self-sustained epileptiform field potentials, referred to as interictal-like spikes and ictal-like discharges, were observed from the CA3, piriform, and entorhinal areas following perfusion of slices with high-bicarbonate ACSF (Fig. 11*A*–*C*). The interictal spikes featured peak amplitudes of ≥0.5 mV, durations of ≤ 500 ms and incidences of 2–5 events/10 s. The ictal discharges manifested with larger peak amplitudes (≥1 mV), longer durations (mean values of ≥30 s), and less frequent incidences (mean interevent intervals of ≥90 s; Fig. 11*E*). Bath application of phenytoin (50–100 μM for 8–10 min) suppressed CA3 or piriform ictal discharges in 4 or 3 slices of kindled mice examined, whereas CA3 interictal spikes remained detectable in the presence of phenytoin (Fig. 10*A*–*C*). These observations are in line with the previous findings that 4-aminopyramine-induced entorhinal ictal discharges but not interictal events were abolished by other clinically used antiepileptic drugs (carbamazepine, topiramate, and valproic acid) in rat brain slices (D'Antuono et al. 2010).

The propensities to exhibit regional epileptiform field potentials were different between slices of kindled and control mice (Fig. 11*D*). Monitored from the CA3 area, interictal spikes were observed in 23 slices and ictal discharges in remaining 14 slices of kindled mice, whereas only interictal spikes were detected in all 33 slices of control mice. Monitored from the piriform area, ictal discharges were observed in 14/15 slices of kindled mice, whereas only interictal spikes were detected from 3/15 slices of control mice. Monitored from the entorhinal area, interictal spikes or ictal discharges were observed from 5 or 7 of 18 slices of kindled mice, whereas only interictal spikes were observed in 8/15 slices of control mice. The proportion of slices with detected CA3 or piriform ictal discharges was significantly greater in slices of kindled mice than in slices of control mice (Fisher’s exact test, *P* ≤ 0.01; Fig. 11*D*). In addition, while regional ictal discharges appeared at similar times following perfusion of high-bicarbonate ACSF (4.02 ± 0.44, 4.75 ± 0.54, and 4.46 ± 0.98 min for the CA3, piriform and entorhinal areas, respectively), the proportion of slices with detected ictal discharges was significantly greater for the piriform than the CA3 or entorhinal area (Fisher’s exact test, *P* ≤ 0.05; Fig. 11*D*). Together these observations suggest that self-sustained ictal discharges induced by high-bicarbonate ACSF may be a unique feature of slices of extended kindled mice and that the ability to generate such in vitro ictal discharges is not limited to the kindled hippocampal CA3 circuitry.

### Brain Histological Observations

**Figure 12:**
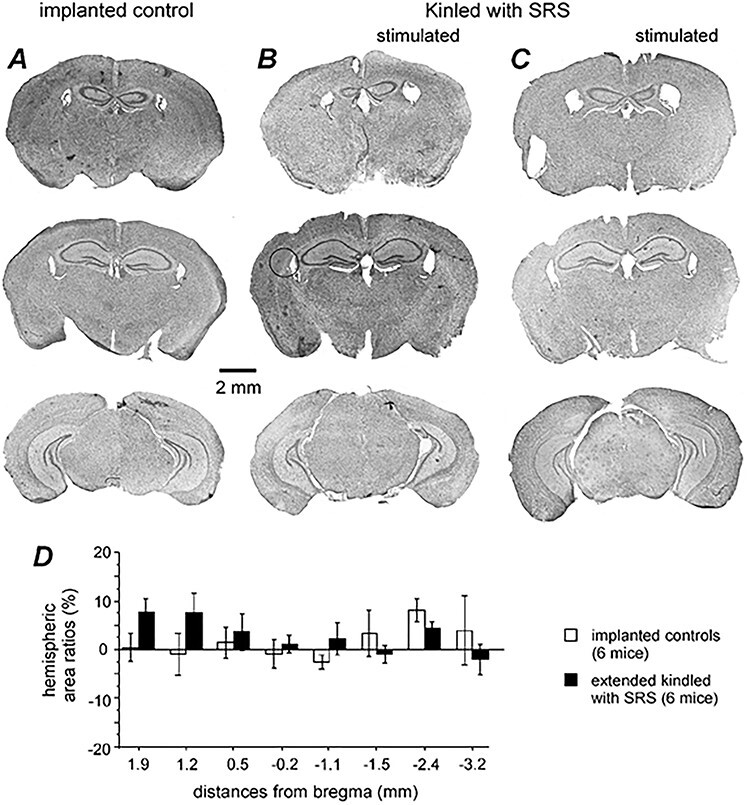
General histological observations from control and extended kindled mice. (*A*–*C*) Images taken from a control mouse (*A*) and 2 extended kindled mice with SRS (*B* and *C*). Cryostat coronal sections (50 μm) were stained with cresyl violet and images were obtained by a slide scanner at ×20 magnification. Kindled hemispheres in (*B* and *C*) indicated. (*D*) Ratios (%) of bilateral hemispheric areas. Hemispheric areas were measured at 8 coronal levels as indicated in *x*-axis. Data were obtained from 6 control and 6 kindled mice. There was no significant group difference at each coronal level tested (Student’s *t*-test or Mann–Whitney *U* test, *P* > 0.05).

We obtained coronal brain sections (50 μm thick) from 8 extended kindled mice with detected SRS and from 6 control mice. These sections were stained with cresyl violet for general morphological assessments. Putative tip locations of implanted electrodes were identified in 13 mice. These locations were approximate to the stereotaxic coordinates of the targeted hippocampal, cortical, entorhinal, or thalamic areas (Supplementary Fig. 1). Gross brain injury unrelated to implanted electrodes, such as structural deformities, cavities, and dark-stained scar tissues as previously observed from mouse models of brain ischemia (El-Hayek et al. 2011; Wang et al. 2015; Wu et al. 2015; Song et al. 2018a), were not observed in these kindled and control mice (Fig. 12*A*–*C*). There was no evident atrophy in the kindled hemisphere as the area ratios of the kindled versus contralateral hemisphere, assessed at 8 coronal levels, were not significantly different between the extended kindled and control mice (*n* = 6 each group, Fig. 12*D*). However, disrupted tissues along putative tracks of implanted electrodes were noted in 3 kindled mice. It is unclear whether such tissue disruption resulted from initial insertions of electrodes and persisted afterward or happened due to electrode withdrawal during brain dissection, or both. Signs of discreet cell injuries, such as shrunken cytoplasmic areas, and dark-stained nuclei and/or cell loss, were variably observed in hippocampal or other areas of kindled mice. Together these observations suggest that extended kindled mice with SRS exhibit subtle-to-moderate cell injuries of varied degrees but not gross brain injury.

## Discussion

### Similar SRS Progression in Mice With Different Electrode Implantations

In our present experiments, each mouse was implanted with 2 pairs of bipolar electrodes. One pair of electrodes was placed in the kindled hippocampus and another pair in an ipsilateral or contralateral unstimulated forebrain structure. The latter structure was alternated in 5 groups of mice and targeted the contralateral hippocampus, ipsilateral/contralateral parietal cortex, ipsilateral piriform cortex, contralateral dorsomedial thalamus, or ipsilateral entorhinal cortex. In response to extended hippocampal kindling, the numbers of stimuli, cumulative evoked ADs, and motor seizures needed to induce SRS were largely comparable in mice of the hippocampal-hippocampal, hippocampal-cortex, hippocampal-piriform, and hippocampal-thalamus groups. While data from mice in the hippocampal-entorhinal group were not included for group comparison due to a small sample size, overall, these observations suggest that electrode implantation approach used in our experiments is not a major determining factor of SRS progression.

The SRS progression we observed in mice seems to be different from that previously observed in rats. In the rat model of extended kindling, the numbers of stimulations required to induce SRS were in a range of 92–508 (mean 348; Pinel and Rovner 1978a, 1978b), 192–277 (Michael et al. 1998), or 174–281 (Brandt et al. 2004). SRS emergence was also observed following 300 stimulations (Milgram et al. 1995) or after 90–100 evoked stage 5 motor seizures (Sayin et al. 2003). Cumulative durations of evoked ADs to SRS were ranged from 13 761 to 27 405 s (Brandt et al. 2004). The measures from rats are apparently greater than those we observed from mice (Fig. 1*C*,*E*), raising a possibility that intracranial electrode implantation in the small mouse brain may promote SRS progression. However, we conducted hippocampal kindling in mice of 11–13 months old, and the previous studies kindled various structures (amygdala, hippocampus, entorhinal cortex, caudate, olfactory bulb, or performant path) in adult rats (initial body weights of 200–500 g). It is conceivable that multiple experimental variables, including animal species/strains, animal ages, kindling sites, and intracranial electrode implantation, may influence SRS progression in the rodent model of extended kindling.

### SRS With Predominant Expression of Stage 3–5 Motor Seizures

We assessed spontaneous motor seizures using the Racine 0–5 stages modified for mice (Racine 1972; Reddy and Rogawski 2010; Reddy and Mohan 2011) because of similar behavioral appearances of spontaneous and evoked motor seizures in hippocampally kindled mice. Briefly, spontaneous stage 3–5 motor seizures manifested with bilateral forelimb clonus, rear, and fall; spontaneous stage 0–2 motor seizures were recognized by behavioral arrest, chewing/facial movement, and head nodding/unilateral forelimb clonus. SRS that presented stage 3, 4, or 5 motor seizures were frequently observed from individual mice in the hippocampal-hippocampal, hippocampal-cortex, hippocampal-piriform, and hippocampal-thalamus groups, whereas SRS that expressed stage 0, 1, or 2 motor seizures were more frequently observed from mice in the hippocampal-thalamus group. Overall, proportions of SRS with expression of stage 0–2 motor seizure were greater in the hippocampal-thalamus group than in other groups. These observations suggest that hippocampally kindled mice may predominantly exhibit stage 3–5 spontaneous motor seizures and that perturbation of the dorsomedial thalamic circuitry by chronic electrode implantation may influence the expression pattern of spontaneous motor seizures. While the underlying mechanisms remain to be explored, the latter may be in line with the modulatory roles of the dorsomedial thalamic circuitry previously observed from other seizure models (Bertram et al. 2001; Sloan et al. 2011; Bertram 2014).

There were limitations and complications in our assessments of spontaneous motor seizures. We analyzed spontaneous motor seizures that were accompanied with EEG discharges, but we did not detect spontaneous motor seizures by video analysis alone. As such we might have underestimated spontaneous motor seizures such as myoclonic jerks associated with interictal spikes previously described in extended kindled rats (Pinel and Rovner 1978a, 1978b; Michael et al. 1998; Brandt et al. 2004). In addition, we used only a webcam to monitor motor activity for each mouse. It was difficult to recognize stage 0–2 motor seizures when the mouse was not faced to the webcam, which might have rendered more errors in scoring stage 0–2 than stage 3–5 motor seizures. Moreover, we did not isolate EEG-video monitoring from environmental noises and used dim lighting during the light-off period. These factors might have influenced the incidence and severity of spontaneous motor seizures detected in our experiments. In rats that underwent extended amygdala kindling and then combined EEG-video monitoring (12 h per day for 7 consecutive days), 3–12 SRS events with expression of stage 4–5 motor seizures were observed from the monitoring periods (Brandt et al. 2004). In our experiments, extended kindled mice exhibited 3–11 SRS events per day and SRS with expression of stage 3–5 accounted ≥85% of total SRS observations in most mice. If our observed stage 3–5 motor seizures are comparable to the stage 4–5 motor seizures recognized in kindled rats (Brandt et al. 2004), then the expression of generalized spontaneous motor seizures appeared to be more frequent in hippocampally kindled mice than in amygdala-kindled rats (Brandt et al. 2004). The abovementioned experimental confounds may also partly explain this apparent difference.

### SRS Manifested With Concurrent EEG Discharges in Forebrain Areas

We monitored spontaneous EEG discharges from the kindled hippocampus and an unstimulated forebrain structure in mice of the 5 implantation groups. In nearly all SRS events with decipherable EEG signals in both implanted areas, discharges were found to coexpress in the 2 corresponding structures. Most discharges began with the LVF signals irrespective of the targeted structures and associated motor seizures, and the LVF onsets of corresponding regional discharges appeared concurrently in all corresponding discharge events examined. Collectively, these observations suggest that concurrent discharges of the kindled hippocampus and corresponding unstimulated forebrain structures are a predominant electrographic feature of SRS in hippocampally kindled mice.

We used local differential recording through twisted bipolar wire electrodes to sample “local” EEG signals while attenuating influences of remote signals. The discharges simultaneously recorded from the kindled hippocampus and corresponding unstimulated structure were generally different in waveform. Additionally, “focal” spikes before or following discharges were observed from the kindled hippocampus but not from corresponding forebrain structures. Hippocampal discharges alone were also observed in some of corresponding hippocampal-thalamic recordings. While region-specific signals were observed, caution should be taken when interpreting these observations. EEG signals recorded via fine intracranial electrodes are generally referred to as local field potentials (Buzsáki et al. 2012). Multiple sources can contribute to the local field potentials including synaptic activities, intrinsic ionic currents, electronic interactions via gap junctions among neurons and non-neuronal cells, and ephaptic effects. The waveform, amplitude, and frequency of the local field potentials are dependent upon the proportional contributions of these sources, the distances between these sources and recording site, the geometry and architecture of recorded brain tissue, and the impact of volume conduction (Buzsáki et al. 2012). The latter is particularly pertinent to our present experiments as the volume conduction impact is conceivably greater in the small mouse brain than in larger brains of other animal species. We performed PAC and WPC analyses to explore dynamics of regional discharges and the impacts of volume conduction.

PAC is a type of cross-frequency coupling analysis where the phase of slow oscillations modulates the amplitude of faster oscillations (Tort et al. 2010). PAC has been increasingly used to investigate physiological and pathological brain activities including epileptic discharges (Hyafil et al. 2015). For EEG discharges recorded by subdural electrodes from surgical candidates of epilepsy patients, there were strong PAC between the slow (2–9 Hz) and fast (50–200 Hz) oscillatory signals in middle and later epochs of regional discharges. Such PAC were more prominent in recording sites within than those outside a surgically confirmed seizure onset zoom, suggesting a sensitive marker of epileptogenic circuitries (Weiss et al. 2013; Zhang et al. 2017; Bandarabadi et al. 2019; Grigorovsky et al. 2020). A dynamical and temporal evolution of PAC was noticed for spontaneous regional discharges recorded from kindled mice. The low oscillation whose phase modulates high-frequency oscillations started mainly in 1–4 Hz band for discharge onset and then increased to as high as 16–20 Hz with discharge progression. Although there was no consistent PAC difference in discharge onset, PAC in middle and/or later parts of discharges were distinct between the kindled hippocampus and corresponding unstimulated forebrain structures. Specifically, mean PAC index during discharges were significantly greater or less in the kindled hippocampus than in corresponding parietal cortex, dorsomedial thalamus, or piriform cortex. While these PAC differences are limited in assessing regional epileptogenicity as discharges were monitored from only 2 sites in each mouse, they do suggest that discharges from the kindled hippocampus and corresponding unstimulated forebrain structures differ in oscillatory activities and modulation patterns.

WPC analysis measures possible correlations between 2 signals by assessing their differences in instantaneous phases. Such phase differences can be presented in a time-frequency scalogram that shows temporal phase changes for different frequencies. In comparison to other relevant analyses, WPC allows separation of the effects of amplitude and phase when measuring the relations between 2 simultaneously recorded signals. As fast brain oscillations are typically associated with low amplitudes, WPC is an effective tool for the study of spatiotemporal relationships spanning the frequency domain (Thatcher 2012). WPC analysis revealed temporal phase changes of corresponding regional discharges recorded from kindled mice. In general, an increase in signal phase coherence was not evident for discharge onsets relative to predischarge activities. Phase-locked or near phase-locked signals became appreciable for the early, middle, and/or later epochs of corresponding regional discharges, but these coherent signals expressed with variable lengths and in relative narrow frequency bands in most discharge events analyzed. Such variable and nonuniform expression of coherent discharge signals cannot be fully explained by volume conducted remote signals, assuming the latter through a purely homogeneous and isotropic ohmic environment are phase-locked and more effective for low- than high-frequency signals when recorded from 2 independent sites (Buzsáki et al. 2012). However, volume conduction in the mouse brain particularly during epileptic discharges may not be homogeneous and ohmic, and phase coherence was assessed for only 2 regional discharges in our analysis. As such, it is difficult to determine the contribution of remote volume conducted signals to the observed regional discharges. Considering that these coherent discharge signals were accompanied with dissimilar regional PAC, we postulate that the concurrent discharges observed from the kindled hippocampus and corresponding unstimulated structure represent integrated activities that may largely arise from the local circuitry but encompass signals from surrounding or remote circuitries through volume conduction.

The rodent hippocampus has strong bilateral communications through dorsal and ventral hippocampal commissures (Amaral and Witter 1998). Particularly, CA3 pyramidal neurons have divergent and monosynaptic projections to the contralateral hippocampus (Shinohara et al. 2015). Hippocampal projections to other brain structures, such as the parietal cortex, piriform cortex, dorsomedial thalamus, and entorhinal cortex targeted in our present experiments, are weak and indirect relative to the bilateral hippocampal connections. If the spontaneous discharges are originated primarily from the kindled hippocampus and then spread to other brain structures for generation, the contralateral hippocampus would be expected to receive stronger spread discharge signals and show faster discharge onset relative to other structures. However, regional LVF onsets with similar concurrence were noted between bilateral hippocampal discharges and between hippocampal and cortical discharges or other corresponding regional discharges. It is possible that spontaneous discharges spread from the kindled hippocampus to other unstimulated structures, but that such spread was too fast in the small mouse brain and the onset time lag of corresponding regional discharges was too small to be recognized, possibly obscured by the complex signals immediately preceding the LVF onsets. However, it is difficult to postulate a fast spread of the LVF signals from the kindled hippocampus to other unstimulated structures. Alternatively, spontaneous discharges might arise concurrently in multiple forebrain structures including the kindled hippocampus through a macroscopic epileptic activity, which may be triggered by a common drive yet to be identified (Li et al. 2018). In line with this view, bilateral entorhinal and DG discharges with simultaneous LVF onsets have been demonstrated in a rat model of unilateral intrahippocampal injection of kainic acid (Bragin et al. 2005). Regardless of the underlying mechanisms, corresponding regional discharges with concurrent LVF onsets are a main electrographic feature of SRS in hippocampally kindled mice.

A main technical limitation of our EEG recordings is that only 2 sites were monitored in individual mice. This approach might minimize complications associated with multielectrode implantations in the small mouse brain but disallowed simultaneous detections of discharges from multiple brain structures in each mouse. In addition, 2 recording sites in the small mouse brain are limited in assessing regional discharge spread. Moreover, tip locations of implanted electrodes were histologically examined in a limited number of kindled mice with SRS. While data collected from mice in the 5 implantation groups are supportive of the concurrent expression of forebrain regional discharges, further works that simultaneously record EEG signals from multiple brain structures and histologically depict recording sites in individual mice are required to characterize the temporal relation of regional discharges in our model.

### Epileptiform Discharges Induced Brain Slices In Vitro

We conducted brain slice experiments to examine whether local circuitry activities are altered by extended kindling. In slices perfused with standard ACSF, the amplitudes of evoked CA3 and DG population spikes were not significantly different between kindled and control mice, but DG spike inhibition by paired stimuli (interstimulus intervals of 5–25 ms) was attenuated in kindled mice. Basic intracellular parameters and reversible potentials of pharmacologically isolated IPSCs were largely comparable between CA3 pyramidal neurons of kindled and control mice, but CA3 in vitro SPWs were smaller and less frequent and SPW-related synaptic currents in CA3 pyramidal neurons were more excitatory in kindled mice. However, epileptiform field potentials, which either occurred spontaneous or self-sustained following local afferent stimulation, were not observed from slices of kindled mice when perfused with standard ACSF. While we focused on the CA3 area, our present observations are principally in line with the previous findings in amygdala-kindled rats with SRS (Sayin et al. 2003). In brain slices obtained from extended kindled and control rats, DG spike inhibition by paired stimuli (interstimulus intervals of 15 or 25 ms) was attenuated in kindled rats compared with control rats. In addition, the amplitudes and decay times of evoked IPSCs were decreased in DG granule neurons of kindled rats relative to neurons of control rats, but IPCS reversal potentials and basic intracellular parameters were comparable in both groups of DG neurons. Taking together the previous findings (Sayin et al. 2003) and our present observations, we suggest that local circuitry activities of CA3-DG areas may be altered toward hyperexcitability in extended kindled rats and mice, but such alterations do not lead to the genesis of population epileptiform activities when examined under standard in vitro conditions.

We adopted the approach of high-bicarbonate ACSF (Huberfeld et al. 2011) to examine self-sustained epileptiform activities in slices. Perfusion of slices with high-bicarbonate ACSF induced 2 types of self-sustained epileptiform field potentials. The interictal spikes were observed from slices of kindled and control mice, but the ictal discharges were observed only from slices of kindled mice. The ictal discharges, but not the interictal spikes, were suppressed by phenytoin, which are in line with phenytoin’s effects on EEG activities observed from extended kindled mice (Song et al. 2018b) and with the effects of other clinically used antiepileptic drugs demonstrated in other in vitro models (D'Antuono et al. 2010). In addition, the ictal discharges emerged from the CA3, piriform, and entorhinal areas at similar times following application of high-bicarbonate ACSF, but the propensity of exhibiting ictal discharges was greater in the piriform cortical area relative to the kindled CA3 area. While this difference might be due to the extents of CA3 and piriform circuitries preserved in brain slices and their reactions (see below) to alkaline ACSF, it did suggest that the ability to exhibit self-sustained ictal discharges following exposure to high-bicarbonate ACSF is not limited to the kindled hippocampal CA3 circuitry. Further works are needed to further examine this issue by inducing ictal discharges via different manipulations and in other brain areas.

Major changes in high-bicarbonate ACSF relative to our standard ACSF were a decrease of NaCl (from 125 to 71 mM), increases of KCl (from 3.5 to 6.5 mM) and NaHCO_3_ (from 25 to 80 mM), and an alkaline shift in pH (from 7.35–7.4 to 7.8–7.9) when aerated with 95%O_2_–5%CO_2_. These changes likely induced epileptiform activities by diverse, synergistically acting mechanisms including enhanced ionic and synaptic activities by extracellular alkalization, positive shifts in resting membrane potentials, and the reversal potential of GABAa-IPSPs/IPSCs, and aberrant activities mediated by connexins and pannexin channels (Ruusuvuori and Kaila 2014; de Curtis et al. 2018; Scemes et al. 2019). It is conceivable that the abovementioned ionic and synaptic processes, particularly the glutamatergic activity (Huberfeld et al. 2011), may be altered in extended kindled mice, therefore rendering the expression of self-sustained ictal discharges in slices. While these alterations remain to be investigated in our model, the use of high-bicarbonate ACSF seems a reliable in vitro protocol to reveal heightened ictogenesis of epileptic circuitries in rodent models of chronic SRS.

### No Evident Gross Brain Injury in Extended Kindled Mice Examined

We conducted basic histological assessments in a limited number of extended kindled mice with SRS. Tissue disruptions related to implanted electrodes and discreet cellular injuries of varied degrees were observed, but gross brain injury independent of electrode implantation was not evident in the kindled mice examined. The latter observations are in general agreement with previous studies in extended kindled rats (Pinel and Rovner 1978a, 1978b; Milgram et al. 1995; Michael et al. 1998; Sayin et al. 2003; Brandt et al. 2004). However, we did not perform stereological cell counts and immunocytochemical assessments of GABAergic interneurons as previously demonstrated in extended kindled rats with SRS (Cavazos et al. 1994; Sayin et al. 2003; Brandt et al. 2004). In rats that experienced 150 evoked stage 5 seizures, hippocampal volumes were not reduced but neuronal densities in hippocampal subfields were decreased to 51–81% of control measures. Neuronal densities were also decreased to 76–90% of controls in subfields of the entorhinal cortex but not in the somatosensory cortex (Cavazos et al. 1994). In rats that exhibited SRS following 90–100 evoked stage 5 seizures, the numbers of cholecystokinin-positive GABAergic interneurons in the DG were decreased by 25–76% compared with those in control rats (Sayin et al. 2003; but see Brandt et al. 2004). The decrease in dentate GABAergic interneurons was associated with attenuated dentate spike inhibition by paired stimuli and decreased IPSCs in dentate granule neurons (Sayin et al. 2003). These neuronal losses are thought to resemble pathological findings in human temporal lobe epilepsy (Cavazos et al. 1994; Sayin et al. 2003; Aronica et al. 2017). It is highly likely that hippocampally kindled mice with SRS might suffer similar neuronal loss as previously characterized in extended kindled rats (Cavazos et al. 1994; Sayin et al. 2003). Further works are needed to characterize such neuronal injuries and determine their impacts on progression, incidence, and electrographic initiation of SRS. Considering that bilateral EEG discharges with simultaneous LVF onsets were observed from rats following unilateral intrahippocampal injection of kainic acid (Bragin et al. 2005), it is conceivable that complex mechanisms beyond the initial local brain injury may underlie generalized seizures in rodent models of chronic SRS. In this context, the concurrent regional EEG discharges we observed from extended kindled mice with SRS are unlikely explained by local brain injuries.

## Summary

The main objectives of our present experiments were to detail electrographic features of SRS in the mouse model of extended hippocampal kindling. By intracranial recordings from the kindled hippocampus and different unstimulated forebrain structures in individual mice, we found that EEG discharges with LVF onsets occurred almost simultaneously in corresponding recording sites in nearly all SRS detected and that regional discharge durations were largely unrelated to the severities of the associated motor seizures. By examining local circuitry activities in brain slices, we found that alkaline ACSF induced ictal-like discharges in the CA3, piriform, and entorhinal areas of kindled mice but not control mice, and that the piriform had greater propensity than the kindled CA3 area to generate such in vitro discharges. Together, these in vivo and in vitro observations are supportive of the hypothesis that epileptic activities involving a macroscopic network may generate concurrent discharges in forebrain areas and initiate SRS in hippocampally kindled mice. It is our hopes that our present experiments may help further investigations that examine kindling-induced SRS in mouse models of neurological diseases (Sabetghadam et al. 2020).

## Notes

*Conflict of Interest*: The authors declare that the research was conducted in the absence of any commercial or financial relationships that could be construed as a potential conflict of interest.

## Funding

Ontario Brain Institute (Epilepsy Research Program), Natural Science and Engineering Research Council of Canada (RGPIN 2015); Canadian Institute of Health Research (NSB 2019-2024).

## Supplementary Material

hip-cor26_v4414_stage_5_tgab004

hip-piriform_4_v1702_stage_4_tgab004

hip-thalamus_27_v5144_stage_3_tgab004

Supplementary_data_Jan_14_2021_tgab004
