# Supplementary material for: Electrographic Features of Spontaneous Recurrent Seizures in a Mouse Model of Extended Hippocampal Kindling
Source: Cereb Cortex Commun. 2021 Jan 22;2(1):tgab004. doi: 10.1093/texcom/tgab004 (PMC8152854; doi:10.1093/texcom/tgab004)
Supplement: Supplementary_data_Jan_14_2021_tgab004 [file supplementary_data_jan_14_2021_tgab004.zip › Supplementary_data_Jan_14_2021_tgab004.docx]

**Suppl Table 1**. Times of 24-hour EEG-video monitoring and numbers of spontaneous recurrent seizures (SRS) detected for individual mice in the five implantation groups.

|  | monitoring time (hours) / number of SRS | | | | |
| --- | --- | --- | --- | --- | --- |
| mice  ID | hippocampus-hippocampus | hippocampus-cortex | hippocampus- piriform | hippocampus-thalamus | hippocampus-entorhinal |
| 1 | 47 / 5 | 117.5 / 39 | 94 / 52 | 304.5 / 31 | 70 / 19 |
| 2 | 282 / 66 | 106 / 17 | 117 / 40 | 423 / 224 | 118 /14 |
| 3 | 141 / 34 | 82.4 / 6 | 47 / 25 | 564 / 471 | 82.3 / 16 |
| 4 | 18 / 0 | 359 / 84 | 94 / 23 | 352 / 344 |  |
| 5 | 305 / 82 | 141 / 26 | 35 / 14 | 117 / 12 |  |
| 6 | 141 / 44 | 188 / 28 | 23.5 / 10 | 354 / 514 |  |
| 7 | 36 / 0 | 400 / 73 | 70 / 20 | 117 / 29 |  |
| 8 | 15 / 0 | 235 / 19 | 47 / 16 | 117 / 34 |  |
| 9 | 12 / 0 | 235 / 63 | 47 / 16 | 47 / 5 |  |
| 10 | 8 / 0 | 35 / 4 | 58 / 22 | 341 / 240 |  |
| 11 | 47 / 0 | 258 / 8 | 47 / 19 | 47 / 25 |  |
| 12 | 28.2 / 0 | 141 / 19 |  | 282 / 68 |  |
| 13 |  | 23 / 0 |  | 188 / 82 |  |
| 14 |  | 58.5 / 6 |  | 94 / 1 |  |
| 15 |  | 47 / 18 |  |  |  |

Only SRS with decipherable EEG discharges in both corresponding regional recordings and identifiable motor behaviors in video analysis were accounted for each mouse. For mice without such accounted SRS, ≥5 discharge events from the kindled hippocampus or unstimulated structure with or without identifiable motor seizures were observed from each mouse. Large numbers of SRS were observed from 4 mice in the hippocampus-thalamus group as SRS with stage 0-1 motor seizures occurred frequently and consecutively in these mice.

**

Suppl Fig 1**. Histological examinations of implanted electrodes. Data collected from 10 extended kindled mice and 3 control mice. Putative tip locations of implanted electrodes were inspected in coronal sections (50 µm) stained with cresyl violet. **A**-**D**, schematic presentations of coronal brain sections at indicated distances to bregma. Electrode tip locations indicated by filled circles. **E** and **F**, images taken from 2 kindled mice, showing putative tip locations (filled arrows) of an implanted hippocampal (E) or thalamic (F) electrode.





**Suppl Fig 2.** Measures of low voltage fast (LVF) onset signals. **A**, an example of corresponding EEG activities collected from the kindled hippocampus and ipsilateral piriform cortex. Original signals in a wide frequency band (0.1-1000 Hz) were illustrated. Putative discharge onset and termination indicated by filled and open arrows. Illustrated discharges associated with a stage 3 motor seizure. **B**, LVF onset signals in A were expanded. **C**, traces illustrated in B were treated a band-pass filter of 2-200 Hz. Dotted lines denoted the time periods in which standard deviations (SD) of corresponding regional signals were obtained. **D**-**G**, SD values were similarly obtained from individual mice in four implantation groups (≥20 discharge events per mouse). SD of LVF signals were normalized as percentiles of preceding (baseline) signals. **#**, data from 3-5 mice (3-5 discharges per mouse) were pooled together.





**Suppl Fig 3**. EEG traces collected from a mouse in the hippocampal-thalamus implantation group. Original signals in a frequency band of 0.1-1,000 Hz were illustrated. Filled or open arrows denoted putative discharge onset or termination. **A**, discharges associated with a stage 2 motor seizure and displayed concurrent LV onsets. **B-C**, two events were recorded during consecutive stage 0-2 motor seizures. Note that hippocampal discharges began with repetitive incremental spikes and that these hippocampal discharges were not associated evident discharge signals in corresponding thalamic recordings.


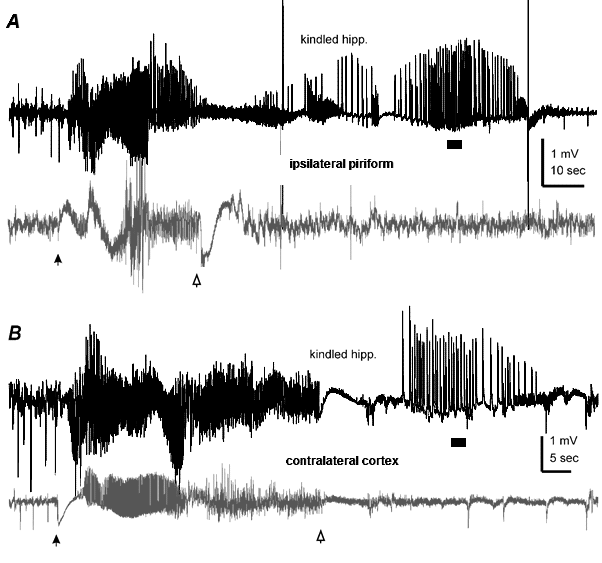


**Suppl Fig 4**. “Local” hippocampal spikes observed from the kindled hippocampus. EEG traces collected from two kindled mouse. Original signals in a frequency band of 0.1-1,000 Hz were illustrated. Filled or open arrows denoted putative discharge onset or termination. **A**, discharges collected from the kindled hippocampus and ipsilateral piriform cortex and associated with a stage 3 motor seizure. **B**, discharges recorded from the kindled hippocampus and contralateral parietal cortex and associated with a stage 4 motor seizures. Note in A and B large amplitude spikes following hippocampal discharges (top, denoted by filled rectangles) but not corresponding piriform or cortical discharges (bottom).


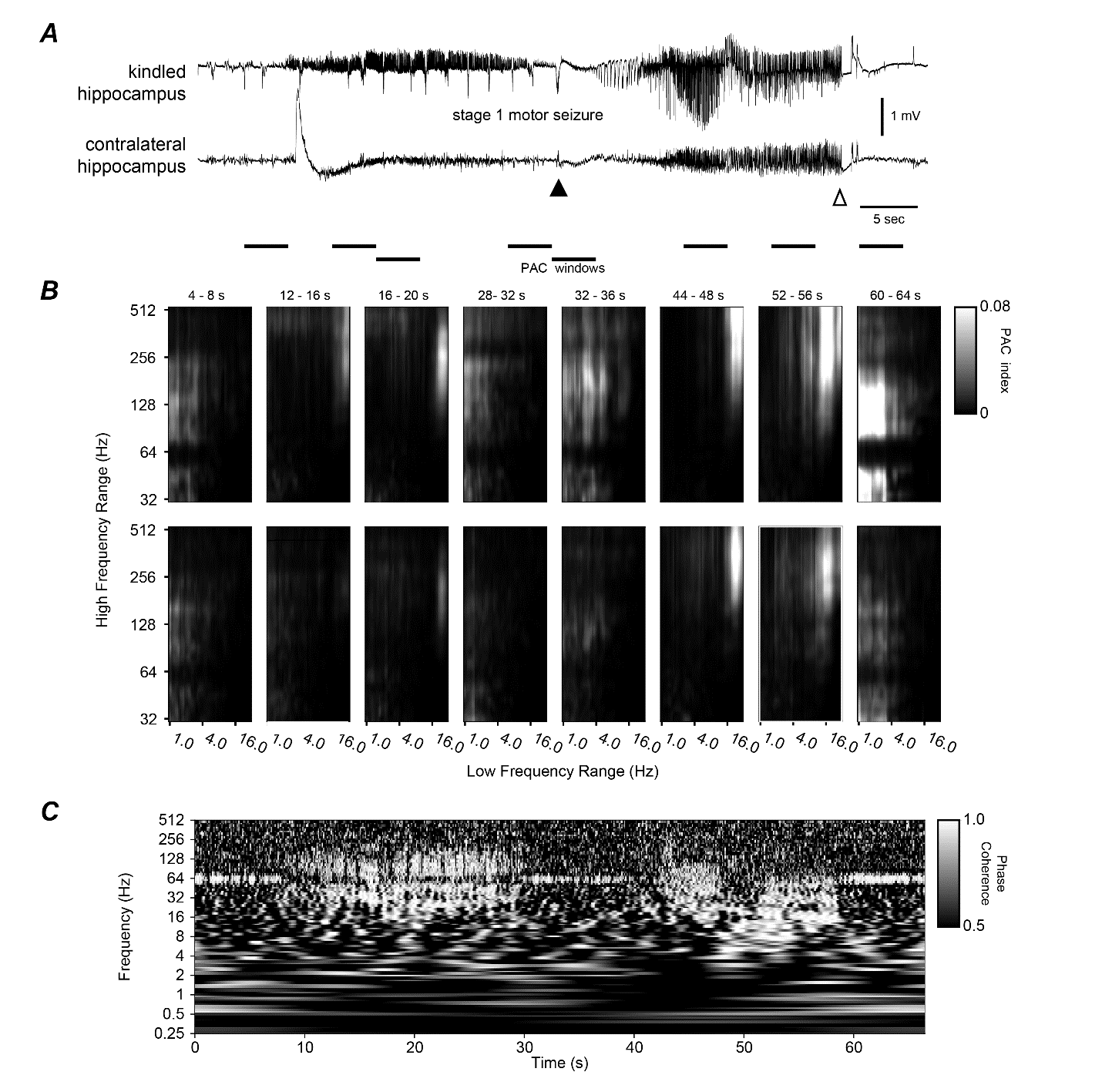
**Suppl Fig 5.** Bilateral hippocampal discharges, corresponding Phase-Amplitude Coupling (PAC), Wavelet Phase Coherence (WPC) analyses. **A**, original EEG activities in a frequency band of 0.1-1000 Hz were illustrated. Putative discharge onset or termination denoted by a filled and open arrow. Discharges associated with a stage 3 motor seizure. Note low-amplitude spike activity in the kindled hippocampus prior to discharge onset. **B**, PAC for the kindled (top) and contralateral (bottom) hippocampal activities. Eight sequential windows matched horizontal bars in A and corresponding times indicated for individual windows. The low frequency range used for the phase information was 1–30 Hz and the high frequency range used for the amplitude information was 32–512 Hz, with increments on a logarithmic scale. Note in windows 6-7 stronger PAC between 20-25 Hz (X axis) and 128-512 Hz (Y axis) signals in the top (kindled hippocampus) than in bottom (contralateral hippocampus) panels. **C**, WPC plot for corresponding regional EEG activities illustrated in A. WPC was applied to each wavelet central frequency from 0.25 – 512 Hz with increments on a logarithmic scale and window size proportional to 8 cycles of each frequency. Scale 1 indicated a phase lock. Note phase-locked or near phase-locked appearing in the 43-58 sec time stamps and in a frequency range of 10-128 Hz.


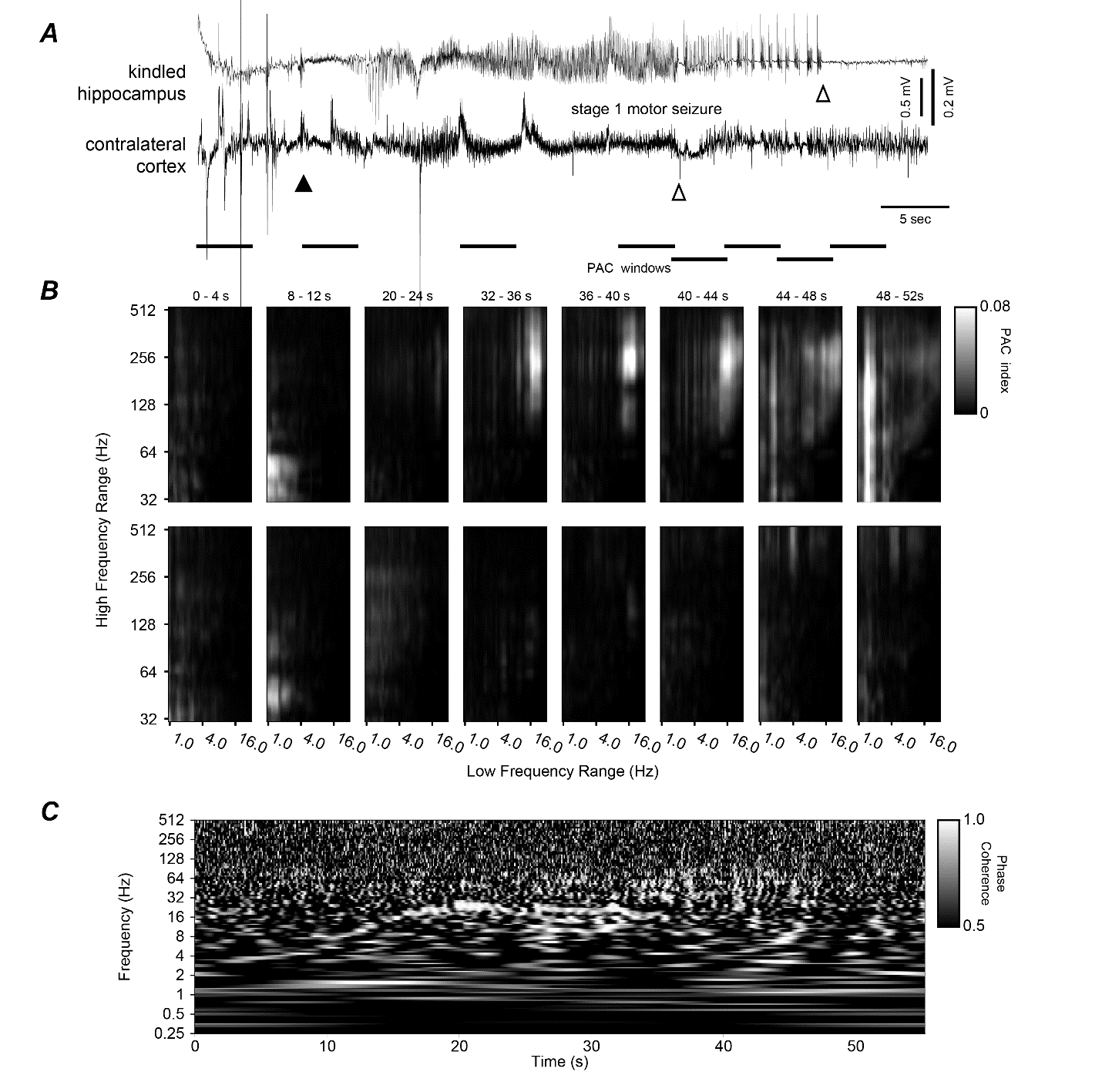


**Suppl Fig 6.** Hippocampal-cortical discharges, corresponding PAC, and WPC analyses. **A**, original EEG signals similarly illustrated as Suppl Fig 1. Discharges associated with a stage 1 motor seizure. Note different times of regional discharge termination. **B**-**C**, WPC and PAC plots similarly arranged as in Suppl Fig 1. Note windows 4-6 of B there were stronger PAC between 16-25 and roughly 100-500 Hz signals in the top (kindled hippocampus) than bottom (unstimulated cortex) panels. Also note phase-locked or near phase-locked discharge signals appearing in the 16-33 sec time stamps and in a frequency range of roughly 10-32 Hz. These coherent signals were accompanied by dissimilar regional PAC in windows 3-4 of B.


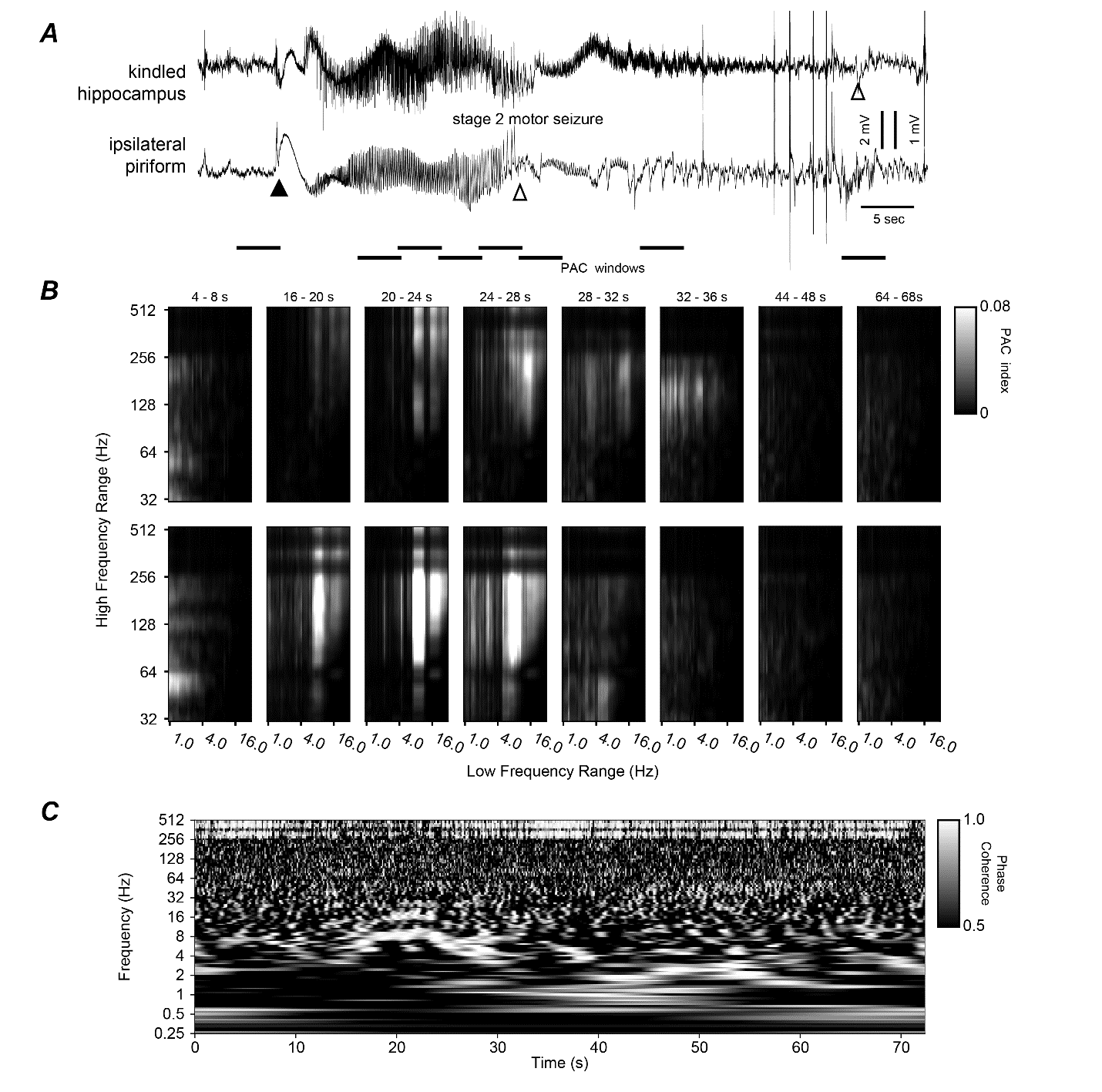


**Suppl Fig 7**. Hippocampal-piriform discharges, corresponding PAC, and WPC analyses. **A**, original signals similarly illustrated as in Suppl Fig 1. Discharge associated with a stage 4 motor seizure. Note different termination times of regional discharges. **B-C**, WPC and PAC plots similarly arranged as in Suppl Fig 1. Note in windows 3-5 of B there were stronger PAC between 8-12 Hz and roughly 64-256 Hz signals in the top (hippocampus) than bottom (piriform) panels. Also note phase-locked or near phase-locked discharge signals appearing in the 15-32 sec time stamps and in a frequency range of roughly 4-16 Hz. These coherent signals were accompanied by dissimilar regional PAC in windows 2-5 of B.


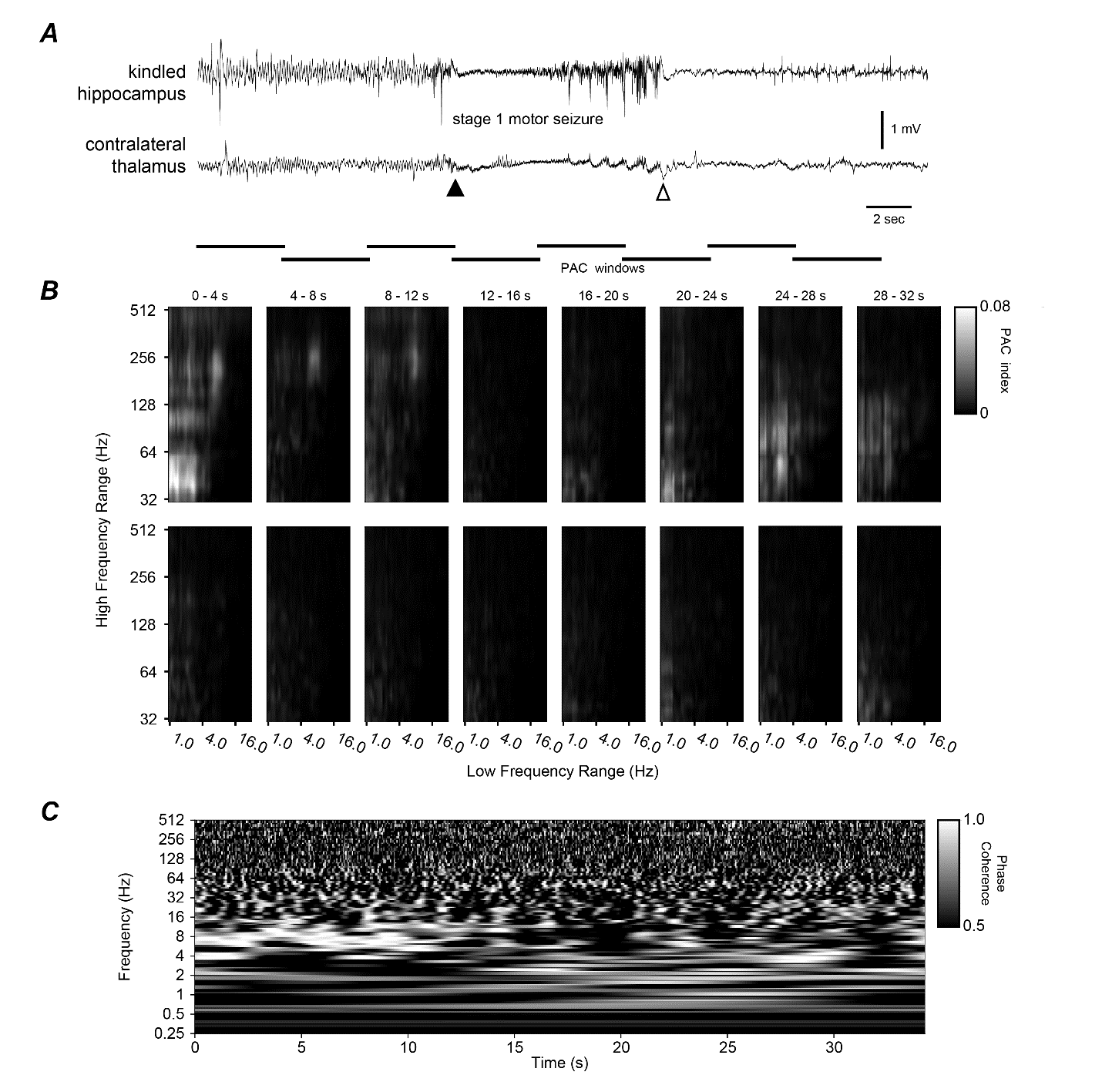


**Suppl Fig 8**. Hippocampal-thalamic discharges, corresponding PAC, and WPC analyses. **A**, original EEG signals similarly illustrated as in Suppl Fig 1. Discharges associated with a stage 1 motor seizure. Note brief discharges with LVF onsets for both recording sites. **B-C**, WPC and PAC plots similarly arranged as in Suppl Fig 1. Note in windows 4-5 of B there were weak but different regional PAC that matched brief regional discharges. Also note in C phase-locked or near phase-locked discharge signals were noticeable before (0-12 sec time stamps) but not evident during discharge (13-22 sec time stamps).


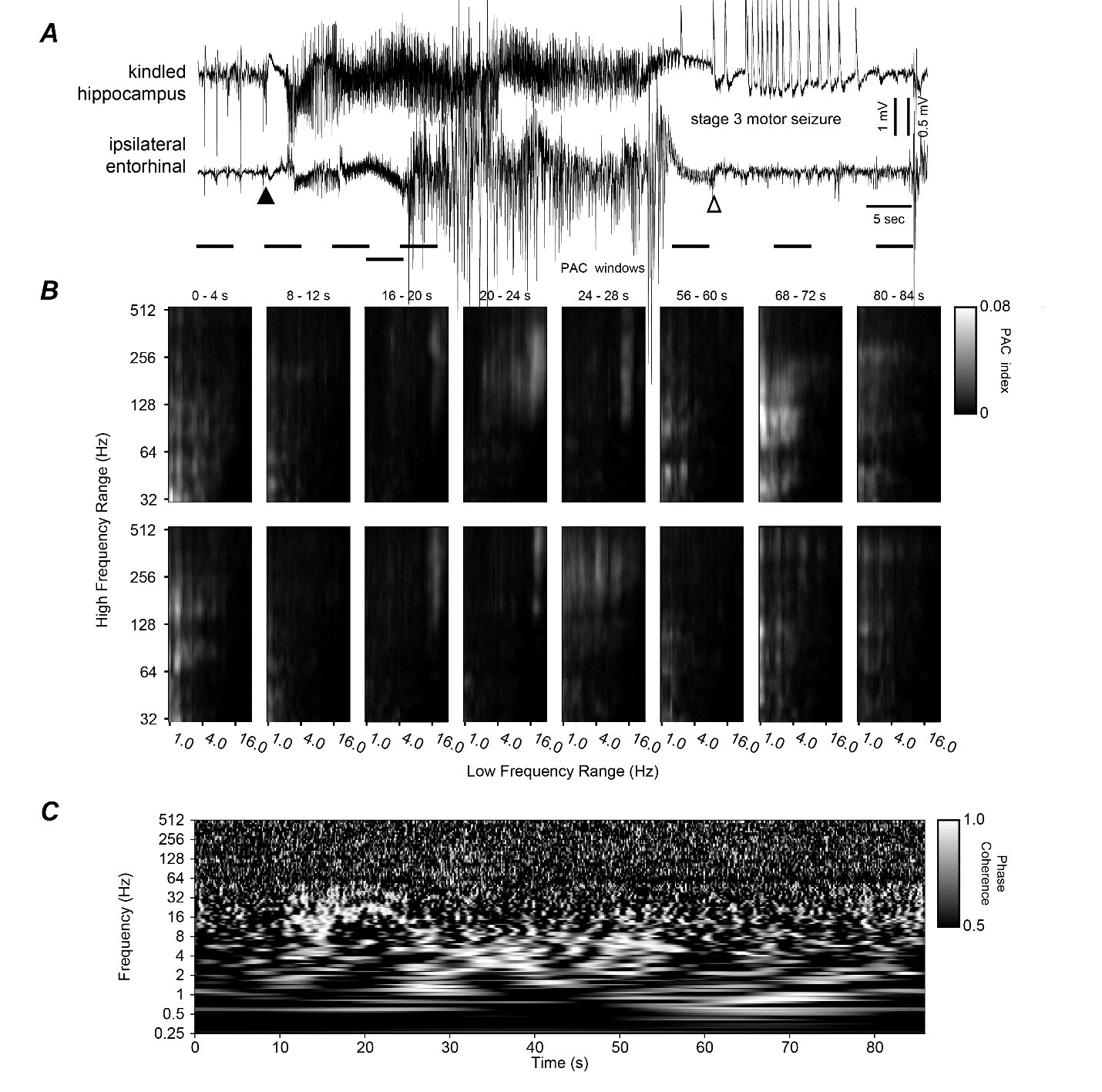


**Suppl Fig 9**. Hippocampal-entorhinal cortical discharges, corresponding PAC, and WPC analyses. **A**, original EEG signals similarly illustrated as in Suppl Fig 1. Discharges associated with a stage 3 motor seizure. Note artefacts in the middle part of discharges and large spikes following hippocampal discharge. **B-C**, WPC and PAC plots similarly arranged as in Suppl Fig 1. Note in windows 3-7 of B there were weak but different regional PAC. Also note in C phase-locked or near phase-locked discharge signals appearing the 11-25 sec time stamps and in a frequency range of roughly 8-50 Hz.





**Suppl Fig 10**. EEG traces collected from an extended kindled mouse. Discharges were recorded simultaneously from the kindled (top) and contralateral hippocampus (bottom). **A**, discharges were collected about 13 hours after termination of hippocampal kindling. **B**, discharges collected 9 weeks later. Putative discharge onsets denoted by filled arrows. **C**, discharge onsets in A and B were expanded. Note discharges began with hypersynchronous (HYP) at left and with low voltage fast (LVF) signals at right.

**
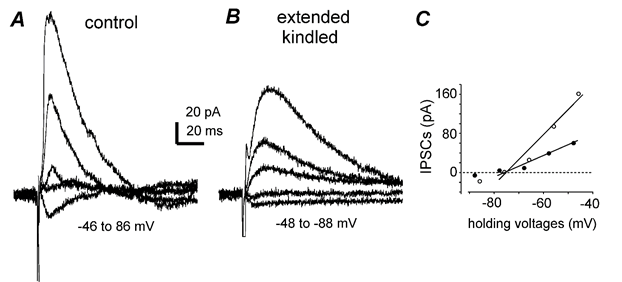
**

**Suppl Fig 11**. Evoked IPSCs and estimated IPSC reversal potentials for CA3 pyramidal neurons. **A**-**B**, voltage-clamp recordings made from CA3 pyramidal neurons of a control mouse and an extended kindled mouse with SRFS. Holding potentials were -46 to -86 mV or -48 to -88 mV (10 mV per step) for the control (A) or kindled (B) neuron. IPSCs were evoked by local stimulations in the presence of a general glutamate receptor antagonist kynurenic acid (2.5 mM). **C**, IPSC peak amplitudes were plotted vs. holding voltages for the control and kindled responses in A (open circles) and B (filled circles). Solid lines were computed via linear regression function (r^2^≥0.92). The voltage with estimated zero current was considered as the reversal potential of IPSCs. IPSC reversal potentials were similarly estimated for 7 CA3 pyramidal neurons from 3 control and 6 neurons from 4 kindled mice (-72.4±1.6 mV and -71.6±2.4 mV, p=0.480, Student t test).

**Suppl video 1**. A spontaneous stage 5 motor seizure captured from a mouse in the hippocampal-cortex group. EEG discharges associated with the motor seizure were shown in Fig 5A. Video arranged with seizure starting around the 55 sec time stamps.

**Suppl video 2**. A spontaneous stage 4 motor seizure captured from a mouse in the hippocampal-piriform group. EEG discharges associated with the motor seizure were shown in Fig 6A. Video arranged with seizure starting around the 16 sec time stamps.

**Suppl video 3**. A spontaneous stage 3 motor seizure captured from a mouse in the hippocampal-thalamus group. EEG discharges associated with the motor seizure were shown in Fig 7A. Video arranged with seizure starting around the 30 sec time stamps.
